# Precise Characterization of Genetic Interactions in Cancer via Molecular Network Refining Processes

**DOI:** 10.3390/ijms222011114

**Published:** 2021-10-15

**Authors:** Jinmyung Jung, Yongdeuk Hwang, Hongryul Ahn, Sunjae Lee, Sunyong Yoo

**Affiliations:** 1 Division of Data Science, College of Information and Communication Technology, The University of Suwon, Hwaseong 18323, Korea; jmjung@suwon.ac.kr (J.J.); ydhwang@suwon.ac.kr (Y.H.); hrahn@suwon.ac.kr (H.A.); 2 School of Life Sciences, Gwangju Institute of Science and Technology, Gwangju 61005, Korea; 3 Department of ICT Convergence System Engineering, Chonnam National University, Gwangju 61005, Korea

**Keywords:** genetic interaction, molecular networks, refining process, cancer therapeutics

## Abstract

Genetic interactions (GIs), such as the synthetic lethal interaction, are promising therapeutic targets in precision medicine. However, despite extensive efforts to characterize GIs by large-scale perturbation screening, considerable false positives have been reported in multiple studies. We propose a new computational approach for improved precision in GI identification by applying constraints that consider actual biological phenomena. In this study, GIs were characterized by assessing mutation, loss of function, and expression profiles in the DEPMAP database. The expression profiles were used to exclude loss-of-function data for nonexpressed genes in GI characterization. More importantly, the characterized GIs were refined based on Kyoto Encyclopedia of Genes and Genomes (KEGG) or protein–protein interaction (PPI) networks, under the assumption that genes genetically interacting with a certain mutated gene are adjacent in the networks. As a result, the initial GIs characterized with CRISPR and RNAi screenings were refined to 65 and 23 GIs based on KEGG networks and to 183 and 142 GIs based on PPI networks. The evaluation of refined GIs showed improved precision with respect to known synthetic lethal interactions. The refining process also yielded a synthetic partner network (SPN) for each mutated gene, which provides insight into therapeutic strategies for the mutated genes; specifically, exploring the SPN of mutated *BRAF* revealed *ELAVL1* as a potential target for treating *BRAF*-mutated cancer, as validated by previous research. We expect that this work will advance cancer therapeutic research.

## 1. Introduction

The identification of cancer-*essential* genes specific to a certain mutated gene, also referred to as synthetic lethal interactions, is crucial for establishing therapeutic strategies and understanding the mechanisms of cancer [1]. Inhibiting genes that are synthetically lethal to a certain mutation would kill cancer cells harboring such mutations while sparing normal cells, which could facilitate precision medicine [2,3]. For example, the *PARP1* gene has been proven to be an essential gene specific to mutated *BRCA* (i.e., synthetically lethal to mutated *BRCA*), and the use of the *PARP1* inhibitor *olaparib* was approved for treating *BRCA*-mutated ovarian cancer [4]. On the other hand, cancer *suppressor* genes specific to a certain mutated gene also provide opportunities for cancer therapeutics. Cancer cells harboring a certain mutation can be killed via the activation (or upregulation) of the suppressor genes specific to the mutation [5], even though this approach is more challenging than inhibiting essential genes. In this study, a cancer*-essential* (*suppressor*) gene *K* specific to a mutated gene *Q* is referred to as a *sensitive* (*resistant*) genetic interaction (GI) between a mutated gene *Q* and a gene *K*, as introduced in Lord et al. (refer to Section 4.2 for detailed description) [6].

GIs are typically characterized by loss-of-function perturbation using CRISPR and RNAi [7]. Statistical analysis of cancer growth after knocking out/down genes by CRISPR/RNAi in multiple cancer cells yields quantitative assessments of GIs [8]. To date, many groups have systematically identified GIs at the genome scale by performing high-throughput loss-of-function screening on a panel of cancer cell lines using CRISPR or RNAi [9,10,11]. However, CRISPR and RNAi techniques have their own limitations and yield considerable false positives in the identification of GIs. For example, knockout by CRISPR sometimes induces cell death mediated by the DNA damage response irrespective of target gene inhibition. In addition, the RNAi approach involves off-target effects that silence the mRNA molecules of unintended targets [7]. These are probably the reasons that few GIs have been reproduced across multiple studies [6]. In addition, large-scale multiple testing may be another factor contributing to the false positives. Multiple testing is necessary to analyze loss-of-function screening data for thousands of genes performed on cells containing thousands of mutations, but it inherently leads to considerable false positives [12].

Multiple previous studies have proposed new computational strategies to decrease potential false positives when characterizing GIs by CRISPR/RNAi screening. Lord et al. identified robust GIs that were reproduced across four distinct loss-of-function screenings, including two RNAi and two CRISPR screenings [6]. In addition, Jerby-Arnon’s group assessed not only essentiality profiles by shRNA-based loss-of-function screenings but also coexpression profiles and coinactivation information based on genomic signatures for detecting synthetic lethal interactions [13]. Furthermore, from the initial synthetic lethal pairs acquired by large-scale gene knockout experiments, Lee et al. selected the clinically relevant synthetic lethal pairs whose co-inactivation lead to better patient survival [14].

In this study, two kinds of processes were newly devised and applied to decrease false prediction in characterizing GIs by applying constraints that consider actual biological phenomena. First, loss-of-function data of nonexpressed genes were excluded in characterizing GIs, under the assumption that they would not affect cell systems. We noticed that one out of six of the analyzed genes in this study was nonexpressed, and knockout/knockdown of nonexpressed genes theoretically should not influence any cell processes. This means that technical defects, such as off-target effects, exist if their depletion scores are not trivial. Second, more importantly, the characterized GIs were refined by utilizing molecular networks such as Kyoto Encyclopedia of Genes and Genomes (KEGG) and protein–protein interaction (PPI) network analysis, under the assumption that genes genetically interacting with a certain mutated gene are located adjacently in the networks. The second assumption is derived from the fact that a chemical signal is transmitted through a cell as a series of biochemical events on molecular networks, which ultimately results in a cellular process, such as cell proliferation or apoptosis (i.e., signal transduction). Thus, if gene *K* genetically interacts with mutation *Q* by altering a certain pathway, the genes connected to gene *K* in the pathway are likely to genetically interact with mutation *Q*. In other words, if gene *K* genetically interacts with mutation *Q* and the genes connected to gene *K* on the networks do not, the GI between mutation *Q* and gene *K* could be a false interaction. In this study, genes genetically interacting with a mutated gene *Q* are referred to as synthetic partners (SPs) of gene *Q.*

In this study, GIs were characterized by incorporating the three kinds of profiles, namely mutation, loss of function, and expression profiles, in the DEPMAP database and exploring the molecular networks via KEGG and PPI network analysis. Here, the two kinds of processes introduced above were newly employed to decrease the occurrence of false predictions. In addition, the refining process (RP) based on molecular networks yielded a synthetic partner network (SPN) for each mutated gene, which provided good insight into the mechanism or therapeutics of cancer. The results were evaluated for the previously known synthetic lethal interactions in the two datasets from MiSL and synlethDB, which allows improved precision in most comparisons. We expect that this work will reduce false GI characterizations and provide assistance in cancer research. The abbreviations used in this study are listed in Table 1.

## 2. Results

### 2.1. Strategy Overview

**Figure 1:**
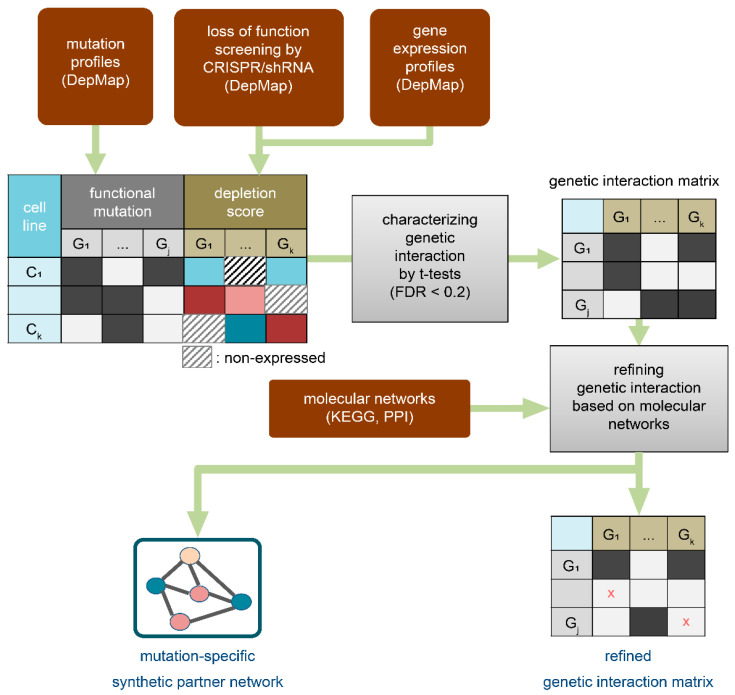
Strategy overview. The genetic interaction (GI) between a mutated gene Q and a gene K was characterized when the knockout/knockdown of gene K statistically caused cancer death or proliferation in cells harboring mutated gene Q. To quantitatively estimate this, a *t*-test was applied to depletion scores from loss-of-function screening (CRISPR and shRNA) between cells with mutated and normal genes, where the depletion scores of nonexpressed genes were excluded. In addition, the characterized GIs were further refined by incorporating the KEGG or PPI networks based on the assumption that genes genetically interacting with a certain mutated gene (i.e., synthetic partners of a certain mutated gene) are located adjacent in the networks. The objective of these two processes was to diminish potential false predictions. As a result, we obtained a refined set of GIs and a synthetic partner network (SPN) for each mutated gene.

The mutation profiles of 18,000 genes across 1747 cell lines were obtained from DepMap, and they were marked as functional mutations if deleterious, such as frame shifts, stop codon deletions, and missense mutations. Out of 18,000 genes, the 4000 recurrently mutated genes, i.e., functionally mutated in more than 3% of the considered cell lines, were analyzed in this study. Next, the depletion scores by loss of functions from CRISPR knockout screening (16,000 genes across 769 cell lines) and shRNA knockdown screening (6000 genes across 702 cell lines) were individually acquired from DepMap. The GI between a mutated gene *Q* and a gene *K* were characterized when the knockout/knockdown of gene *K* statistically caused cancer death or proliferation in cells harboring mutated gene *Q*, which was executed by applying t-tests to the depletion score of gene *K* between cells with mutated gene *Q* and normal gene *Q* (FDR < 0.2). Here, we newly employed the excluding and refining processes to diminish false GIs. First, the depletion scores of nonexpressed genes were excluded in the t-tests, with the assumption that knockout/knockdown of nonexpressed genes would not affect cell systems. Second, the characterized GIs were further refined by incorporating the KEGG or PPI networks. The assumption was that SPs of a certain mutated gene are located adjacent in the networks. The refining process also provides an SPN for a certain mutated gene, which can be used to research the mechanism or therapeutic strategy of the mutated gene (Figure 1). The characterized GIs were evaluated for previously known synthetic lethal interactions in the two datasets from MiSL and synlethDB.

### 2.2. The Characterized GIs

Among 75,000,000 assessments in the CRISPR screenings, 1740 GIs were identified (FDR < 0.2) with the exclusion process for nonexpressed genes (refer to Section 4.2 for detailed description). However, 1623 GIs were characterized (FDR < 0.2) without the exclusion procedure. The exclusion procedure removed the 167 potential false positives and augmented the 284 potential true positives. In the same manner, among 35,000,000 assessments for the shRNA screenings, 1389 and 1459 GIs were determined (FDR < 0.2) with and without the exclusion procedure, respectively.

**Figure 2:**
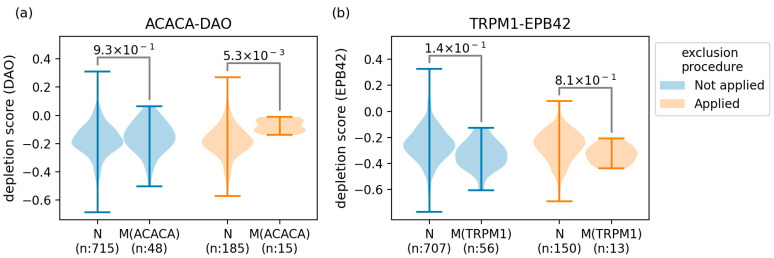
Violin plots of the depletion scores for the GIs whose significance was largely inconsistent according to the use of the exclusion procedure. Depletion scores of the knocked-out gene for normal and mutated cells were violin-plotted for the case where the exclusion procedure was applied or not. (**a**) The resistant GI between mutated *ACACA* and *DAO* was characterized only when the exclusion procedure was applied. (**b**) The sensitive GI between mutated *TRPM1* and *EPB42* was characterized only without the exclusion procedure. The numbers assigned to a pair of violin plots indicate FDRs of *t*-tests. N: normal cells (cells not harboring the mutation), M: mutated cells (cells harboring the mutation), n; number of depletion scores.

For a better understanding of the effect of the exclusion procedure, the depletion scores were illustrated as violin plots for the GIs whose significance was largely inconsistent according to the use of the exclusion procedure (Figure 2). As depicted in Figure 2, a resistant GI between mutated *ACACA* and *DAO* was identified only when the exclusion procedure was applied. On the other hand, a sensitive GI between mutated *TRPM1* and *EPB42* was identified only when the exclusion procedure was not applied, indicating that it could be a potential false positive. Notably, we observed a decreased number of depletion scores for the case where the exclusion procedure was applied. This is because the procedure ignores depletion scores of nonexpressed genes when performing *t*-tests. As a result, 1740 (418 sensitive and 1322 resistant) and 1389 (480 sensitive and 909 resistant) GIs were characterized from CRISPR and shRNA screenings with the exclusion procedure, and these GIs are referred to as ‘original GIs’ in this study. The number of original GIs of each mutated gene is presented in Tables S2 and S4.

### 2.3. The Refined GIs Based on KEGG Network Analysis

The original GIs were further refined based on the directed KEGG networks (refer to Section 4.1.4. for the network construction). To this end, all SPs of the original GIs were mapped to the 1678 nodes on the KEGG networks, which yielded 525 and 417 mapped GIs in the CRISPR and shRNA screenings, respectively. To apply the RP, we further narrowed them down to 162 and 105 GIs of the mutated genes whose number of SPs was two or more, and these GIs were referred to as ‘initial GIs’. For example, in CRISPR screening, the 12 initial GIs of the mutated *BRAF* remained out of its 26 original GIs after mapping on the KEGG networks.

**Figure 3:**
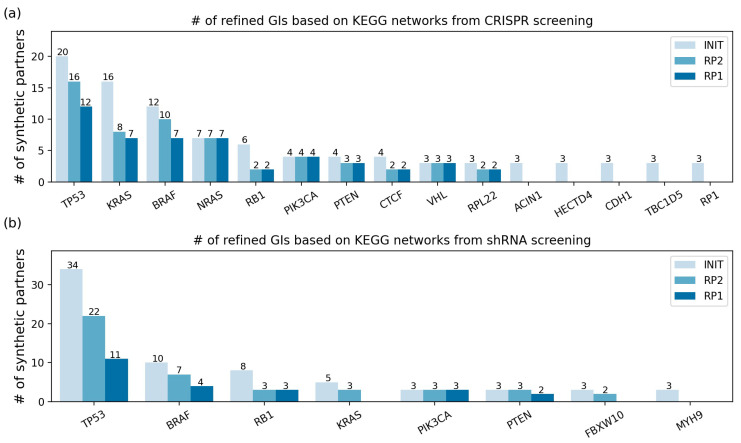
The number of GIs for each mutated gene refined based on KEGG networks from: (**a**) CRISPR screening and (**b**) shRNA screening. Among all results, only the mutated genes that had three or more initial GIs are presented. The *x*-axis indicates each mutated gene. INIT: the initial GIs after mapping on the KEGG networks (no RP was applied). RP2: GIs after applying the RP with a distance of 2 to the initial GIs. RP1: GIs after applying the RP with a distance of 1 to the initial GIs. RP: refining process.

Then, the RP illustrated in Section 4.3 was applied to the initial GIs, and the results are described in Tables S2 and S3 and Figure 3. The number of GIs of each mutated gene is illustrated in Table S2 and Figure 3, and the list of GIs is specified in Table S3. For clarity, Figure 3 and Table S3 contain only the results of the mutated genes whose number of initial GIs is three or more. As a result, the 162 initial GIs in the CRISPR screenings were narrowed down to 73 and 65 GIs by applying the RP with distance 2 (RP2) and the RP with distance 1 (RP1), respectively. In the same manner, the 105 initial GIs in the shRNA screenings were narrowed down to 45 and 23 GIs by applying RP2 and RP1, respectively.

For example, in the CRISPR screening, the application of RP2 to mutated *BRAF* allowed 10 SPs out of the 12 initial SPs, which resulted from filtering out two initial SPs (*SOX9* and *ELAVL1*). The application of RP1 yielded seven SPs by further filtering out three SPs (*MDM2*, *PPP2R2A*, and *SHOC2*). For the *KRAS* mutation, 8 out of the 16 initial SPs (*PTPN11*, *GRB2*, *SOS1*, *NRAS*, *RAF1*, *RRAS2*, *KRAS*, and *ITPR2*) remained with RP2, and *ITPR2* was additionally filtered out by RP1 (Figure 3 and Table S3). On the other hand, in the case of *NRAS* mutation, no SPs were filtered out from the seven initial SPs (*PTPN11*, *GAB1*, *SHOC2*, *GRB2*, *NRAS*, *RAF1*, and *KRAS*) by RP2 and RP1, which means that all initial SPs were adjacent in the KEGG networks. For *RB1* mutation, there were no changes in the remaining SPs between RP1 and RP2, i.e., *SKP2* and *CKS1B* (Table S3). For the five mutated genes (*RP1*, *ACIN1*, *HECTD4*, *CDH1*, and *TBC1D5*) in the CRISPR screening and one mutated gene (*MYH9*) in the shRNA screening, there were no remaining SPs after the RPs, which means that all the initial SPs were distant from each other (Figure 3 and Table S3).

**Figure 4:**
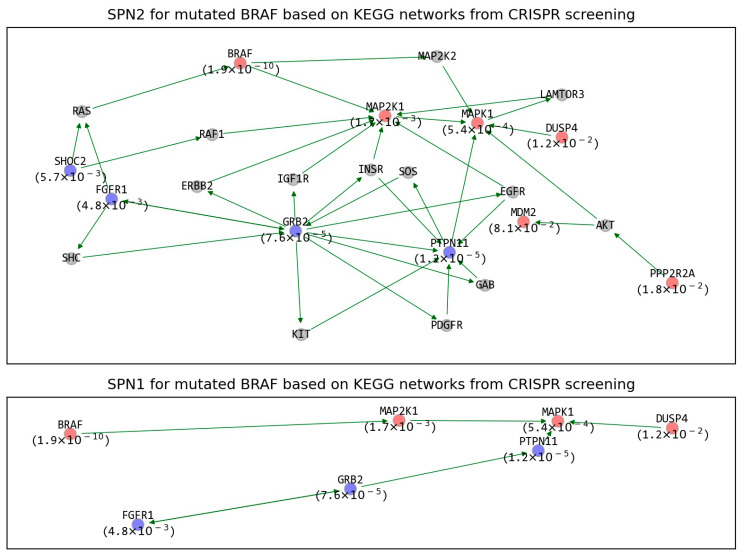
SPN2 and SPN1 for mutated *BRAF* in KEGG networks from CRISPR screening. The red and blue nodes are the SPs associated with GIs sensitive and resistant to mutated *BRAF*, respectively. The gray nodes are not SPs but genes connecting SPs of mutated *BRAF*. SPN2 includes the SPs that are connected to each other within a distance of 2, and SPN1 includes the SPs that adjoin each other. The number in parentheses for each SP indicates its FDR. SPN: synthetic partner network.

An SPN was also constructed for each mutated gene (refer to Section 4.3 for more detail). For example, SPN2 and SPN1 for mutated *BRAF* from CRISPR screening are represented in Figure 4. SPN2 includes 10 SPs (red or blue nodes) and 14 neighbors connecting the SPs (gray nodes). Among the 10 SPs, six are sensitive SPs, i.e., *BRAF*, *MAP2K1*, *MAPK1*, *DUSP4*, *MDM2*, and *PPP2R2A* (represented as red nodes), and four are resistant SPs, i.e., *SHOC2*, *FGFR1*, *GRB2*, and *PTPN11* (represented as blue nodes). Its own inhibition allowed the highest sensitivity (lowest FDR), which is consistent with the fact that BRAF is an oncogene addiction [15]. The seven SPs were directly connected to each other, and the other three SPs (*SHOC*, *PPP2R2A*, and *MDM2*) were indirectly connected via neighbors (such as *RAS* and *AKT*), which makes SPN1 have only seven SPs (Figure 4). Interestingly, *MAPK1* and *PTPN11*, which are contiguous SPs, present opposite types of GIs (sensitive and resistant) on the mutated BRAF. This can be explained by the fact that *PTPN* negatively regulates *MAPK1* [16]. Exploring SPNs can provide new therapeutic strategies. For example, by observing the SPNs of mutated *BRAF*, we noticed that inhibiting the six sensitive SPs or activating the four resistant SPs could be therapeutic strategies for precisely treating *BRAF*-mutated cancer. Furthermore, the 14 neighbors connecting the SPs (such as *AKT*, *SOS*, and *RAF1*) are other candidate therapeutic targets, which might be indicated as false negative results. SPNs for the other mutated genes in Figure 3 are presented in Figure S5.

### 2.4. The Refined GIs Based on PPI

**Figure 5:**
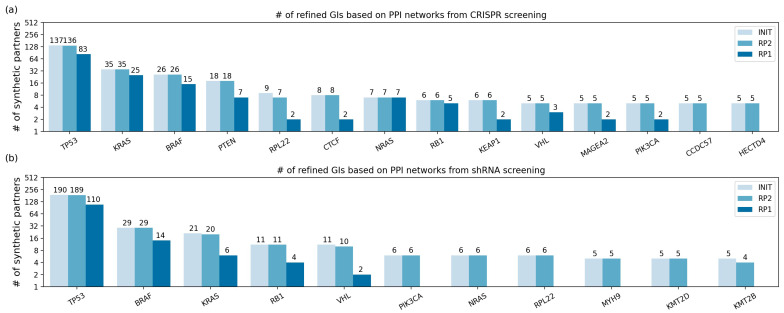
The number of refined GIs based on PPI networks from (**a**) CRISPR screening and (**b**) shRNA screening. Among all results, only the mutated genes with five or more initial GIs are presented. The *y*-axis is log-scale, and the *x*-axis indicates each mutated gene. INIT: the initial GIs after mapping on the PPI networks (no RP was applied). RP2: GIs after applying RP2 to the initial GIs. RP1: GIs after applying RP1 to the initial GIs. RP: refining process.

The original GIs were also refined based on undirected PPI networks. Among the 1740 and 1389 original GIs from CRISPR and shRNA screening, 1699 and 1368 GIs remained after mapping SPs on the PPI networks. Similar to the KEGG network RPs, the RPs were applied to the initial GIs of the mutated genes whose number of SPs was two or more, where the number of initial GIs was 789 and 570 in CRISPR and shRNA screenings. The results are described in Tables S4 and S5 and Figure 5. The number of GIs is illustrated in Table S4 and Figure 5, and the list of GIs is specified in Table S5. For clarity, Figure 5 and Table S5 contain only the results of the mutated genes whose number of initial GIs is five or more. As a result, the 789 initial GIs were narrowed down to 607 and 183 GIs for CRISPR screenings, and the 570 initial GIs were narrowed down to 497 and 142 GIs for shRNA screening with RP2 and RP1, respectively.

We noticed that the initial GIs were the same as the GIs remaining after RP2 for most mutated genes (Figure 5), which means that most of the initial SPs were connected within a distance of two on the PPI networks. This is likely because there are some high-degree nodes in the PPI networks (e.g., 994 degrees for *TP53* and 1621 degrees for *MYC*). However, the number of the remaining GIs was greatly decreased by RP1. For example, in the CRISPR screening, 11 out of 18 SPs of the mutated gene *PTEN* were removed by RP1. Furthermore, for the *CCDC57* and *HECTD4* mutated genes, there were no remaining SPs when RP1 was applied, which indicates that the SPs do not adjoin each other at all.

**Figure 6:**
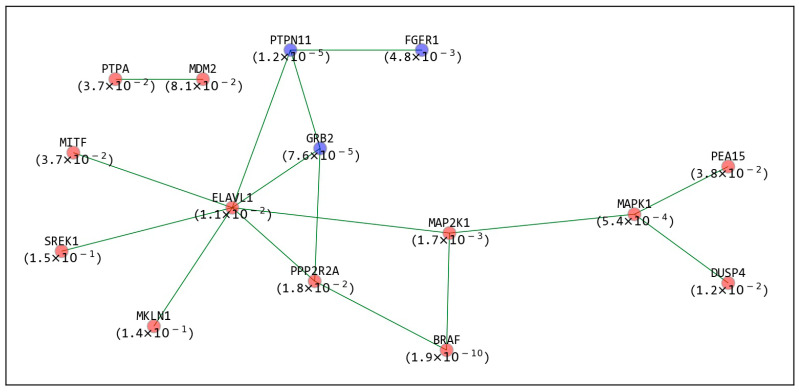
SPN1 for mutated *BRAF* based on PPI networks from CRISPR screening. The red and blue nodes are the SPs associated with GIs sensitive and resistant to mutated *BRAF*, respectively. SPN1 includes only the SPs that adjoin each other. SPN: synthetic partner network.

The SPNs for the PPI networks for each mutated gene were also constructed in the same manner as those for the KEGG networks. For example, SPN1 for mutated *BRAF* from CRISPR screening is depicted in Figure 6. For clarity, SPN2 is not presented as it was complex. The SPN1 consists of the 12 sensitive (red nodes) and three resistant (blue nodes) SPs for mutated *BRAF*. We observed that there was one large single network composed of 13 SPs and one separated edge connecting two SPs, i.e., *PTPA* and *MDM2*. In addition, we noticed that *ELAVL1* was connected to six other SPs (i.e., it had the highest degree in the network), so it can be considered an important therapeutic target. In fact, *ELAVL1* knockdown led to suppression of the proliferation of melanoma cells with mutated *BRAF (V600E)* [17], which is consistent with our results that *ELAVL1* is a sensitive SP for mutated BRAF. Furthermore, *ELAVL1* has been researched as a therapeutic target for various other cancers, such as colorectal, breast, and ovarian cancers [18,19,20]. SPNs for the other mutated genes presented in Figure 5 are depicted in Figure S6, and these networks are quite complicated because the PPIs form densely connected networks.

### 2.5. Evaluation with SynlethDB and MISL

The results were compared to the two kinds of datasets containing synthetic lethal interactions. Synthetic lethal interaction (SLI) is a type of GI between two genes such that simultaneous perturbations of the two genes result in cell death, while a perturbation of either gene alone is not lethal [21]. The first dataset is synlethDB, which is a comprehensive database that contains SLIs collected from biochemical assays, other related databases, computational predictions, and text mining results on human species [21]. All 16,926 SLIs reported in synlethDB were used for the evaluation. The second dataset is the SLIs characterized by the MiSL algorithm, whose underlying assumption is that the synthetic lethal partner of a mutation will be amplified more frequently or deleted less frequently in cancer cells harboring the mutation [22], which yielded 119,548 SLIs in total. Given the definition of SLI, the sensitive GIs were only compared to the two kinds of datasets.

First, the sensitive GIs characterized with the exclusion procedure (SGWEs) and without the exclusion procedure (SGOEs) were evaluated with recall and precision measures. The 418 SGWEs and 389 SGOEs from CRISPR screening were evaluated for each of the two datasets, and their results were compared. In the same manner, 480 SGWEs and 519 SGOEs from shRNA screening were assessed. There were no significant performance differences between SGWEs and SGOEs. In terms of recall, we observed that SGWEs produced slightly better performance for all comparisons, except for shRNA screening evaluated on the MISL datasets (Figure S7). In terms of precision, the SGWEs showed slightly better performance in the shRNA screening and slightly worse performance in the CRISPR screening (Figure S7). Given the evaluation results, we conclude that the use of the exclusion procedure does not yield any significant difference.

**Figure 7:**
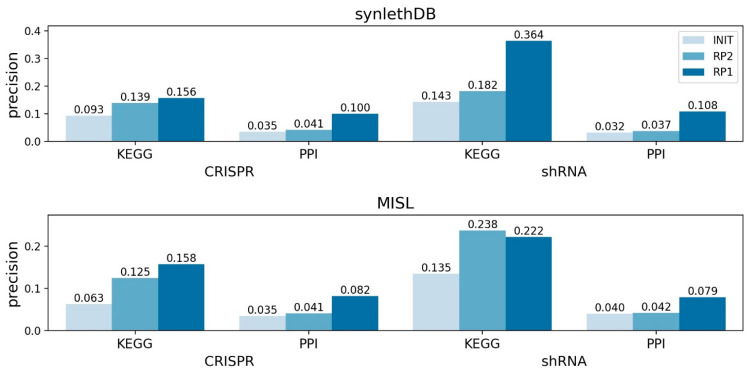
The precision of the sensitive GIs with no RP, RP2, and RP1. The sensitive GIs refined based on KEGG/PPI networks were evaluated with the SLIs in synlethDB and MISL. For most comparisons, RP1 exhibited the highest precision, followed by RP2 and then INIT. INIT: the initial sensitive GIs after mapping on the molecular networks (no RP was applied). RP2: the sensitive GIs after applying RP2 to the initial GIs. RP1: the sensitive GIs after applying RP1 to the initial GIs. RP: refining process. GI: genetic interaction.

Second, the sensitive GIs determined by applying no RP (INIT), RP2, and RP1 were evaluated with recall and precision measures, and the results were compared. In most comparisons, we noticed that recall decreased as the RPs were applied (Figure S8), which was expected because the characterized GIs were narrowed down by applying RP2 and RP1 (i.e., the GIs by RP2 are a subset of those by INIT, and the GIs by RP1 are a subset of those by RP2). However, in precision, we recognized an increasing trend in the order of INIT, RP2, and RP1 (Figure 7) in most comparisons. One exception is the evaluation with MISL for the sensitive GIs from shRNA screening refined by KEGG network analysis, where the precision of RP2 was higher than that of RP1 (Figure 7). According to the evaluation results, we conclude that the precision is enhanced by applying the RPs devised in this study.

We observed that the GIs mapped on the KEGG and PPI networks (i.e., the initial GIs) had higher precision than those before applying the mapping process (i.e., the original GIs). With the original sensitive GIs, the precision ranged from 0.019 to 0.022 for the four comparisons (Figure S7); on the other hand, with the initial sensitive GIs, the precision ranged from 0.032 to 0.093 for the eight comparisons (Figure 7). The fact that many cancer-related genes are included in the KEGG and PPI networks could be one of the reasons for the better performance. In addition, we noticed that the precision was higher for the sensitive GIs refined on the KEGG networks (0.063~0.364) than those refined on the PPI networks (0.032~0.108) (Figure 7). This is because the KEGG networks contain manually curated interactions that are considerably researched and well established.

## 3. Discussion and Conclusions

As one of the applications of the SPNs, comparing SPNs between KEGG and PPI networks of a certain mutated gene can reveal its therapeutic potential in more detail. For example, SPN1 based on the PPI network for mutated *BRAF* from CRISPR screening contained 15 interactions among 15 SPs (Figure 6), while SPN1 based on the KEGG network contained 6 interactions among 7 SPs (Figure 4). We noticed that the edge between *PTPN11* and *MAPK1* in the KEGG network was connected with a path (*PTPN11-ELAVL1-MAP2K1-MAPK1)* in the PPI networks, which is 1 of 1967 possible paths that connects *PTPN11* to *MAPK1* in the PPI within three edges. In other words, we specified a therapeutic path for mutated BRAF that should be more effective because it is supported by both the KEGG and PPI networks.

In addition, comparing SPNs between KEGG and PPI networks provides some interesting observations. For example, we observed that *PPP2R2A* was removed from SPN2 for the KEGG networks (Figure 4) but was contained in SPN1 for the PPI networks (Figure 6), as it directly interacts with other SPs. This was possible because there are much denser interactions in PPI networks than in KEGG networks. On the other hand, as another example, SPN1 for the PPI network for mutated *NRAS* contained seven interactions among seven SPs (Figure S9a). Interestingly, it was identical to SPN1 for the KEGG networks, except for the interaction between *SHOC2* and *NRAS* in the KEGG analysis (Figure S9b). The greater number of edges in the KEGG network was unexpected because PPI networks are more densely connected than KEGG networks. One possible explanation is that there are considerable indirect interactions (i.e., not physical interactions) in KEGG networks.

We also noticed that the GIs determined from CRISPR and shRNA screening were quite different, which is in accordance with findings of Lord’s work [6]. For example, the SPs for mutated *NRAS* in the shRNA screening were largely different from those in the CRISPR screening, where the only common SP is *NRAS* itself (Table S5). Furthermore, for mutated *RPL22*, there was only one shared SP, i.e., *VPS*, between the nine and six SPs from the CRISPR and shRNA screenings (Table S5).

In several previous studies, including the studies of Campbell et al. and Ryan Kelley et al. [23,24], genetic interactions were also generated from high-throughput experiments by incorporating biological networks, such as PPI networks and pathway databases. However, their strategies of using biological networks are somewhat different from this study. For example, in the previous studies, biological networks were mainly applied to a pair of genes, a certain mutated gene and its single SP. On the other hand, in this study, biological networks were applied to all SPs of a certain mutated gene together, except for the mutated gene itself. In other words, we newly considered the connectedness of SPs for each mutated gene on biological networks, which was not addressed in previous work. Therefore, we speculate that our methods could provide new aspects of GI characterization.

The proposed strategy focuses on improving precision in characterizing GIs and thus might fail to identify some true positives. However, in vivo and in vitro experiments, which consume a lot of time and costs, require a handful of therapeutic candidates that are potentially correct. From that perspective, the results of the study could provide assistance to in vivo and in vitro experiments.

There are a few points that could enhance this study, which were not applied due to some limitations. First, we simply assumed that a gene is functionally mutated if associated with 1 of 12 deleterious variant types. This study would have been enhanced if functional alteration was accurately determined for each genetic variant by considering its type, sequence location, and other related information. For example, the dbNSFP database provides a functional prediction of missense mutation in the human genome, based on the collected 54 functional annotation databases [25]. However, it was challenging to find an integrative tool that performs variant-wise functional prediction of all 12 considered variant types. Second, in this study, each cancer type was not regarded in identifying GIs, which is because it is difficult to maintain statistical power due to the limited cell lines when performing cancer type-specific analysis. For reference, we provide the number of cell lines for each cancer type in Figure S10. In this figure, we noticed that the number of all considered cell lines is greater than 600 and 800 in CRISPER and shRNA screening, but that of each cancer type is smaller than 50 in most cases. We believe that plentiful loss-of-function screenings of new cancer cell lines will provide an opportunity to solve this limitation.

In summary, two kinds of GIs, i.e., sensitive and resistant GIs, were identified by exploiting the three kinds of profiles in the DEPMAP database (mutation, loss of function, and expression profiles) and employing molecular networks such as KEGG and PPI networks. In this study, two kinds of processes were newly employed to decrease false predictions. First, knockout/knockdown depletion scores of nonexpressed genes were excluded in characterizing GIs, with the assumption that they would not affect cell systems. Second, the characterized GIs were further refined by utilizing KEGG or PPI networks, with the assumption that genes genetically interacting with a certain mutated gene were adjacent in the networks. When the characterized sensitive GIs were evaluated using the two datasets in MiSL and synlethDB, we noticed that the exclusion process for nonexpressed genes did not produce any improvement in precision and recall. However, the RP for the molecular networks improved precision in most evaluations. The RP also yielded SPNs for each mutated gene, which provides insight into the therapeutic potential of the mutated gene. This work can be improved by exploring larger datasets of loss-of-function profiles for cells containing various functional mutations. In addition, the discovery of unknown interactions in molecular networks will facilitate the development of a more accurate RP. We expect that this work will guide research on cancer therapeutics.

## 4. Materials and Methods

### 4.1. Data Preprocessing

#### 4.1.1. Mutation Profiles

Mutation profiles named ‘CCLE_mutation.csv’ in the DepMap (Broad Institute) database contain ~1,300,000 mutation events for 18,000 genes across 1741 cell lines [26]. A variant type was assigned to each mutation event, and 12 of 20 variant types were considered deleterious, such as de novo start out of frame, frame shift deletion, frame shift insertion, in-frame deletion, nonsense mutation, nonstop mutation, splice site, start codon deletion, start codon insertion, stop codon deletion, stop codon insertion, and missense mutation. A certain gene is determined to be functionally mutated if associated with even one deleterious mutation [22]. The distributions of the number of mutated genes across cell lines are depicted in Figure S1. Out of 18,000 genes, only 4000 genes functionally mutated in more than 3% of the cell lines (i.e., recurrently mutated genes) were analyzed in this study.

#### 4.1.2. Loss-of-Function Profiles

In loss-of-function screening, the depletion score of a gene indicates the number of surviving cells when the gene is knocked out/down [27]. Simply, a low depletion score of a certain gene (i.e., the gene is depleted or underrepresented) means that most cells in which the gene is knocked out/down by CRISPR/RNAi are dead, indicating that the knockout/down of the gene induces cancer death. On the other hand, the high depletion score of a gene (i.e., the gene enriched or overrepresented) implies that most cells in which the gene is knocked out/down by CRISPR/RNAi are alive, supporting that the knockout/knockdown of the gene contributes to cancer proliferation or blocks cancer death [27]. These loss-of-function pooled screenings are typically performed with CRISPR or shRNA genome-wide libraries. In the DepMap database, we acquired the two profiles of depletion scores named ‘Achilles_gene_effect.csv’ (performed in CRISPR knockout screening) [26] and ‘D2_combined_gene_dep_scores.csv’ (performed in shRNA knockdown screening) [28]. The distributions of the number of depleted genes across cell lines are depicted in Figures S2 and S3. After removing all missing values, we obtained depletion scores for 16,000 genes across 769 cell lines from CRISPR screening and 6000 genes across 702 cell lines from shRNA screening.

#### 4.1.3. Expression Profiles

Expression profiles were also obtained from the DepMap database for 19,000 genes across 1305 cell lines [29]. Noticeably, we observed that 4,000,000 records were zero (i.e., nonexpressed) among all 25,000,000 gene expression records. From a gene perspective, 24 genes (such as *CT47A8*, *F8A2* and *USP17L25*) were nonexpressed in all 1305 considered cell lines, and 1637 genes were nonexpressed in more than 1000 of the 1305 cell lines. The distributions of the number of nonexpressed genes across cell lines are depicted in Figure S4.

#### 4.1.4. Network Construction

Two kinds of molecular networks, i.e., KEGG and PPI networks, were constructed to refine the characterized GIs. First, KEGG networks were constructed by integrating 47 KEGG pathways, such as signal transduction pathways, cancer pathways, and cell growth-related pathways (refer to Table S1 for a list of the 47 pathways) [30]. The integrated networks contained 12,617 interactions among 1678 genes. Second, from the BIOGRID database, PPI networks were constructed by integrating protein interactions discovered by the ‘affinity chromatography technology’ or ‘two hybrid’ detection method [31]. The PPI networks provided 373,394 interactions among 18,179 genes. The KEGG networks consisted of directed edges, and the PPI networks consisted of undirected edges.

### 4.2. Characterizing GIs

We considered two kinds of GIs, i.e., sensitive and resistant GIs. First, a sensitive GI between a mutated gene *Q* and a gene *K* is characterized when the knockout/knockdown of gene *K* causes cancer death in the case cells (i.e., cells harboring a mutated gene *Q*) compared to the control cells (i.e., cells with normal gene *Q*). In this case, the depletion scores of gene *K* in the case cells are lower than those in the control cells. Second, a *resistant* GI between a mutated gene *Q* and gene *K* is characterized when the knockout/knockdown of gene *K* causes cancer proliferation or blocks cancer death in the case cells but not in the control cells. In this case, the depletion scores of gene *K* in the case cells are higher than those in the control cells. *T*-tests were used to statistically characterize sensitive and resistant GIs. In more detail, every possible pair between recurrently mutated genes (introduced in Section 4.1.1) and loss-of-function screened genes (introduced in Section 4.1.2) was assessed by applying a *t*-test to the depletion scores of a screened gene between case and control cells, and significant GIs were characterized (FDR < 0.2). Here, we noticed that there were numerous nonexpressed genes in the assessed cells (as introduced in Section 4.1.3), and the depletion scores of the nonexpressed genes were excluded in the *t*-tests. In more detail, depletion scores of a certain gene in the considered cell lines were processed based on the matched expression scores. The expression profiles were also obtained from DepMap database (refer to Section 4.1.3), and DepMap cell IDs (i.e., a primary key) were used for the match process. Here, if the matched expression score is zero (i.e., nonexpressed), its depletion score is ignored in the *t*-test for characterizing genetic interaction. If not, the depletion score is used as it is in the *t*-test (refer to the depletion score matrix in Figure 1). The exclusion procedure was applied to diminish potential false predictions, with the assumption that the knockout/knockdown of nonexpressed genes would not affect cell systems.

### 4.3. Refining GI Based on Molecular Networks

To decrease potential false positives, the refining process (RP) was further applied to the characterized GIs based on the assumptions that the SPs of a certain mutated gene are located adjacently on the molecular networks. Based on the KEGG or PPI networks, the RP was applied to every mutated gene whose number of SPs was two or more. In the process, the SP of a certain mutated gene remained only if there were any other SPs of the mutated gene within a certain distance on the networks, and two kinds of distance (i.e., 1 and 2) were applied. As a result of the RP, a set of refined SPs and SPN were generated for a certain mutated gene. The SPN is the subnetwork containing only the remaining SPs of the mutated gene. SPN*k* indicates an SPN acquired by applying the RP with a distance of *k*. SPNs provide connectivity for the SPs of a certain mutated gene and their associated neighbors, which is advantageous in terms of inferring therapeutic potential of the mutated gene.

## Figures and Tables

**Table 1:** The used abbreviations.

Abbreviation	Meaning
GI	genetic interaction
KEGG	Kyoto Encyclopedia of Genes and Genomes
PPI	protein–protein interaction
RP	refining process
RP1	RP with distance 1
RP2	RP with distance 2
SGWE	sensitive GI characterized with the exclusion procedure
SGOE	sensitive GI characterized without the exclusion procedure
SLI	synthetic lethal interaction
SP	synthetic partner
SPN	synthetic partner network

## Data Availability

Python implementations are available at https://github.com/jmjung83/refined_GIs, accessed on 13 September 2021.

## Supplementary Materials

The following are available online at https://www.mdpi.com/article/10.3390/ijms222011114/s1.
