# Supplementary material for: Precise Characterization of Genetic Interactions in Cancer via Molecular Network Refining Processes
Source: Int J Mol Sci. 2021 Oct 15;22(20):11114. doi: 10.3390/ijms222011114 (PMC8540220; doi:10.3390/ijms222011114)

Figure S1. The distributions of the number of the mutated genes in mutation profiles. (a) The violin-plot for the number of mutated gene in a cell line across 1741 cell lines. The ACH-000999 cell line contains 9698 (the most) mutated gene, and the ACH-001150 cell line contains 12 (the fewest) mutated gene among 18K gene. (b) The histogram of the data presented in (a). (c) The violin-plot for the number of cells involving a mutated gene across 18K genes. The TTN gene is mutated in 1109 (the most) cell lines, and the RPL41 gene is mutated in 1 (the fewest) cell line among 1741 cell lines. The genes mutated in more than 52 (i.e. 3% of 1741) cell lines were only analyzed in this study, allowing 4339 genes (genes above the red line). (d) The histogram of the data presented in (c).

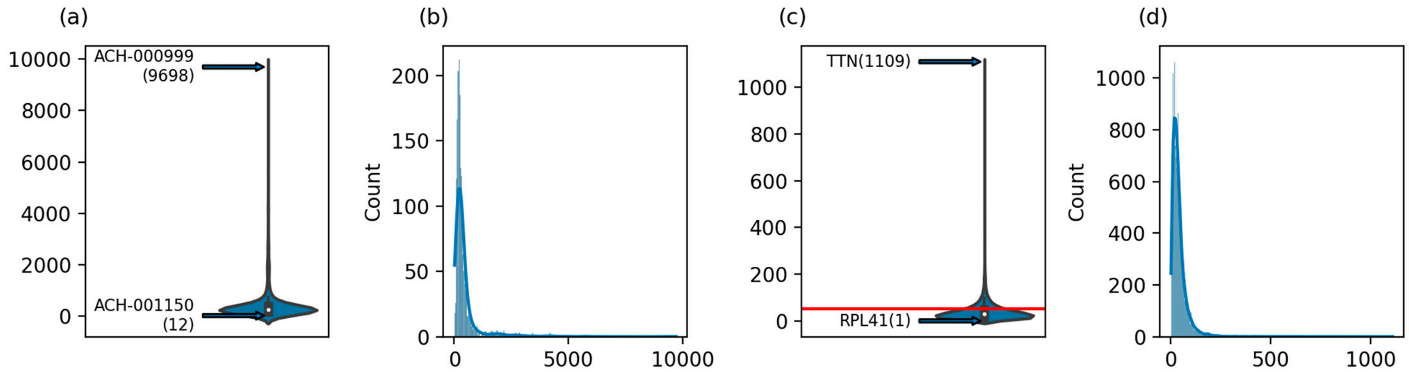

Figure S2. The distributions of the number of the depleted genes in CRISPR screening. (a) The violin-plot for *the number of depleted gene in a cell line* across 1741 cell lines. Here, for simplicity, gene whose depletion score is lower than 0 is regarded as depleted gene (depletion scores are ranged from -4.01 to 6.37). The ACH-001517 cell line contains 10647 (the most) depleted gene, and the ACH-000459 cell line contains 8512 (the fewest) depleted gene among 16K gene. (b) The histogram of the data presented in (a). (c) The violin-plot for *the number of cells involving a depleted gene* across 16K genes. The U2SURP gene is depleted in all 769 (the most) cell lines, and the IGFL1 gene is not depleted in any cell line among 769 cell lines. (d) The histogram of the data presented in (c).

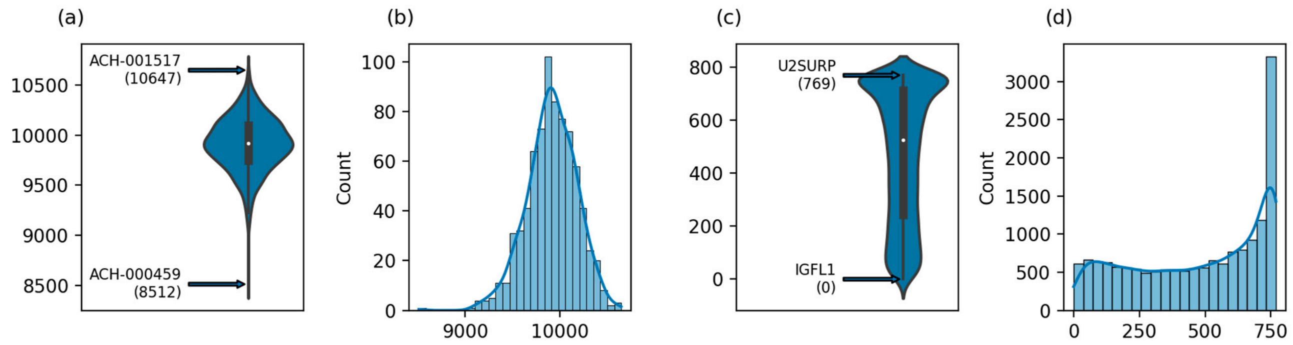

Figure S3. The distributions of the number of the depleted genes in shRNA screening. (a) The violin-p
lot for *the number of depleted gene in a cell line* across 702 cell lines. Here, for simplicity, gene whose d
epletion score is lower than 0 is regarded as depleted gene (depletion scores are ranged from -5.93 to 2.77)
. The ACH-001441 cell line contains 3207 (the most) depleted gene, and the ACH-000869 cell line contains
1340 (the fewest) depleted gene among 6K gene. (b) The histogram of the data presented in (a). (c) The vi
olin-plot for *the number of cells involving a depleted gene* across 16K genes. The CDC23 gene is depleted
in all 702 (the most) cell lines, and the RGPD8 gene is not depleted in any cell line among 702 cell lines.
(d) The histogram of the data presented in (c).

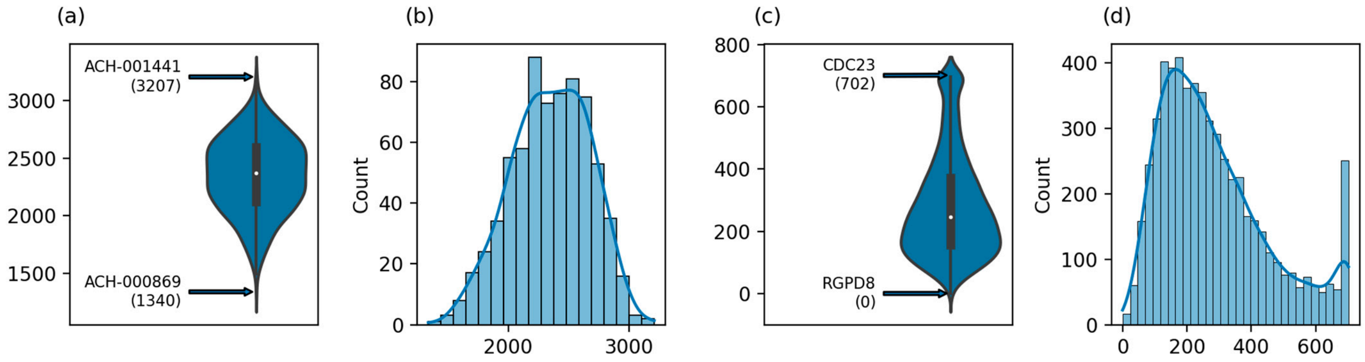

Figure S4. The distributions of the number of the non-expressed genes in expression profiles. (a) The
violin-plot for *the number of non-expressed gene in a cell line* across 1305 cell lines. The ACH-001097 cell
line contains 6298 (the most) non-expressed gene, and the ACH-000682 cell line contains 647 (the fewest)
non-expressed gene among 19K genes. (b) The histogram of the data presented in (a). (c) The violin-plot f
or *the number of cells involving a non-expressed gene* across 19K genes. The CT47A8 gene is non-expresse
d in all 1305 (the most) cell lines, and the MPRIP gene is expressed in all 1305 cell lines. (d) The histogr
am of the data presented in (c).

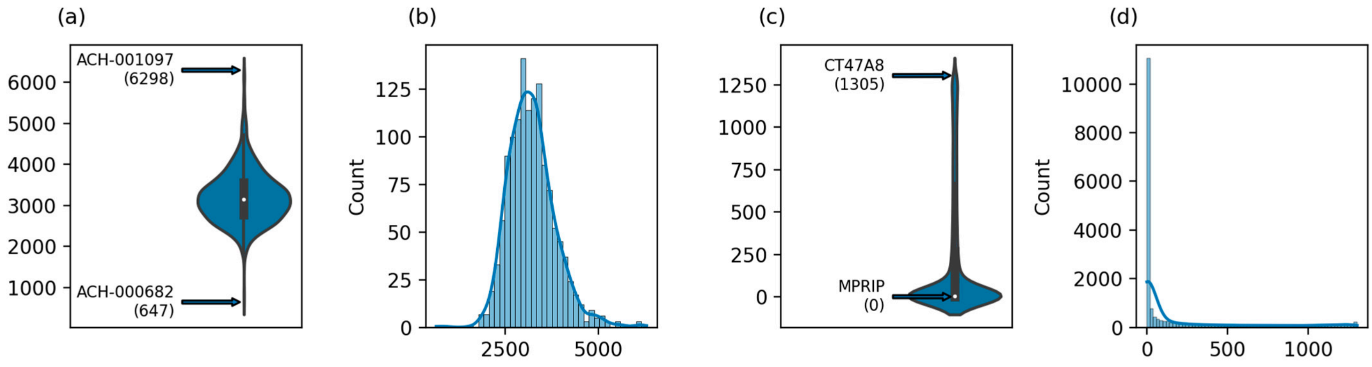

Figure S5. SPNs on KEGG networks from (a) CRISPR and (b) shRNA screenings for the mutated genes in Figure 3.

1. SPNs based on KEGG networks from CRISPR screening

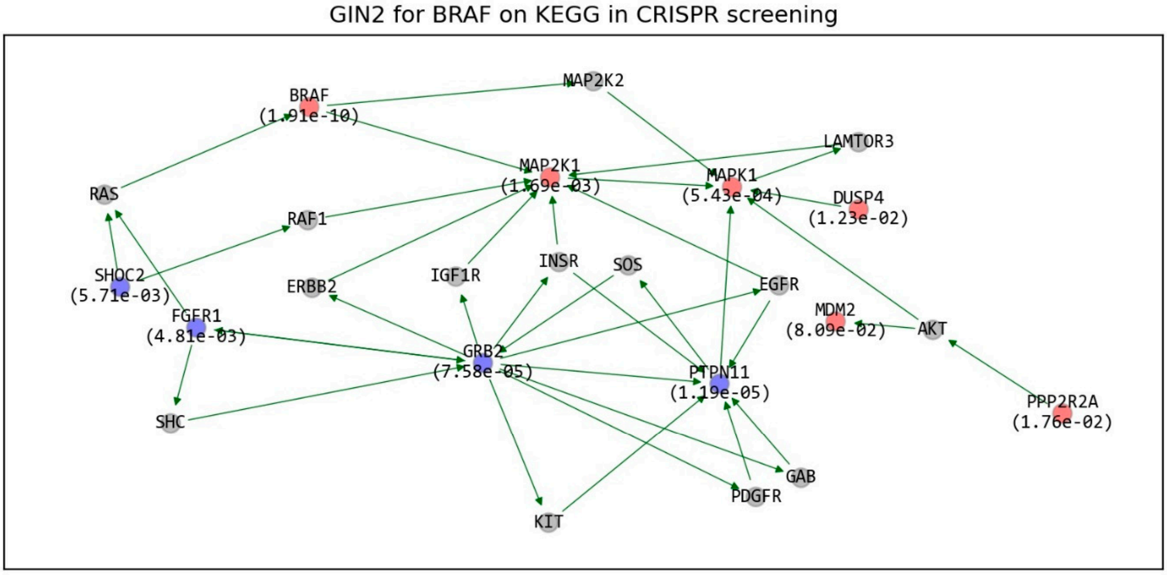

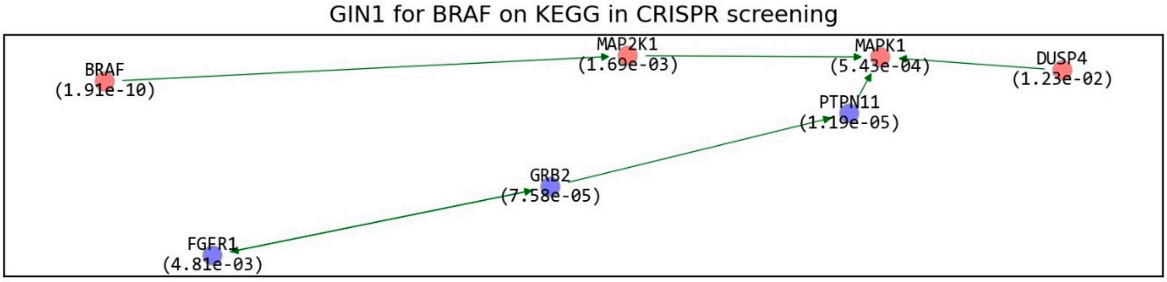

GIN2 for CTCF on KEGG in CRISPR screening

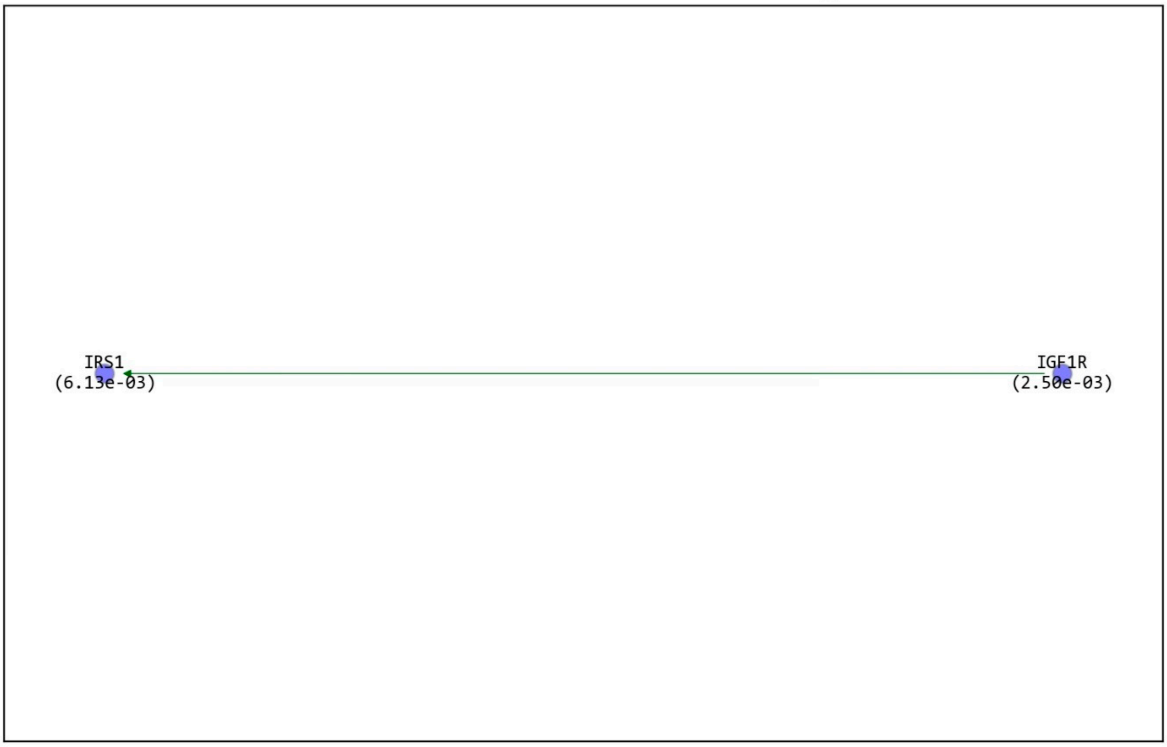

GIN1 for CTCF on KEGG in CRISPR screening

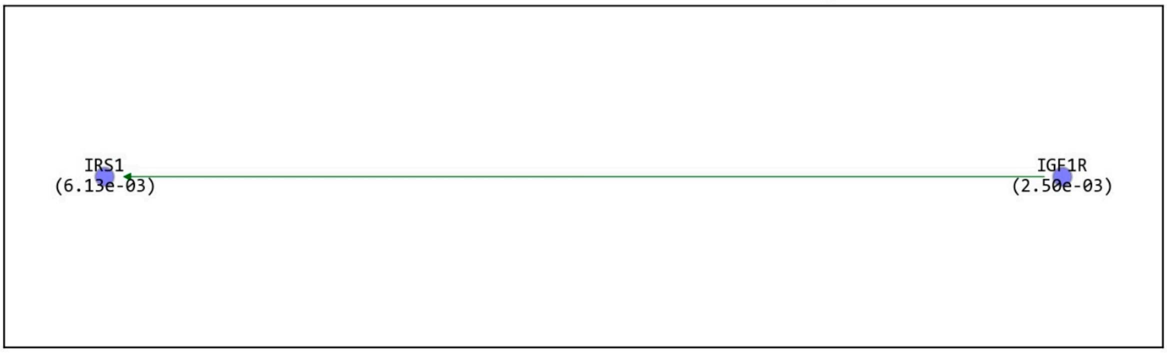

GIN2 for KRAS on KEGG in CRISPR screening

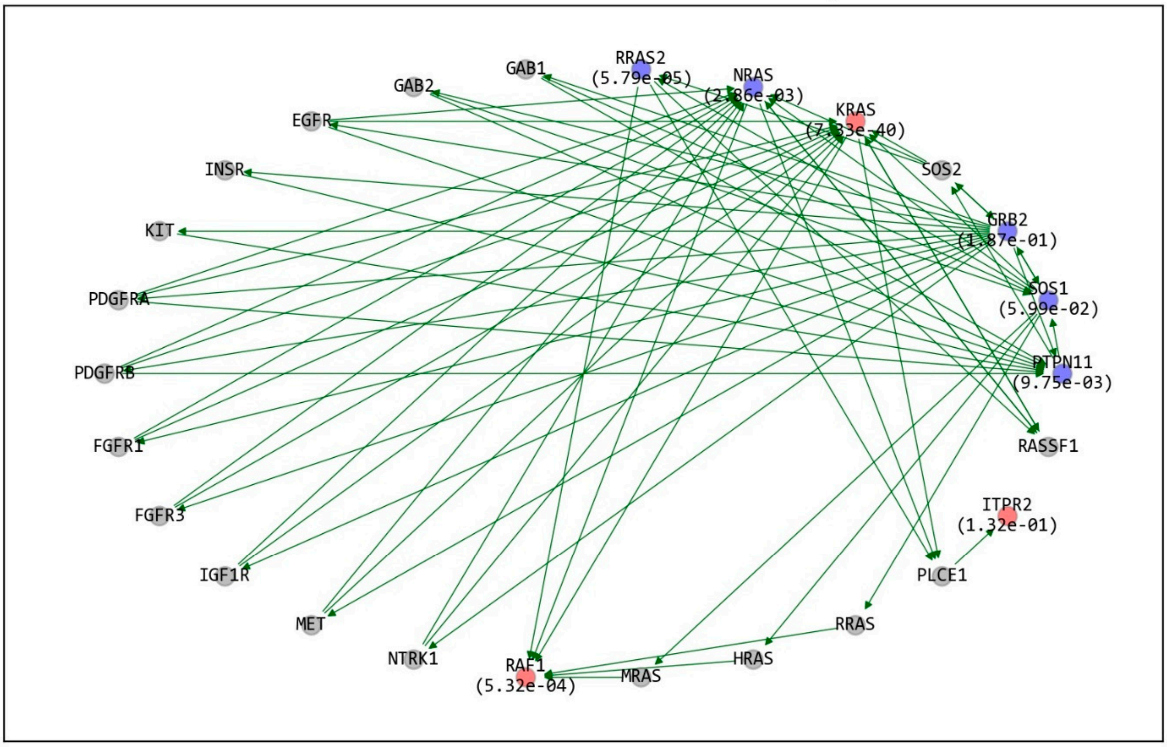

GIN1 for KRAS on KEGG in CRISPR screening

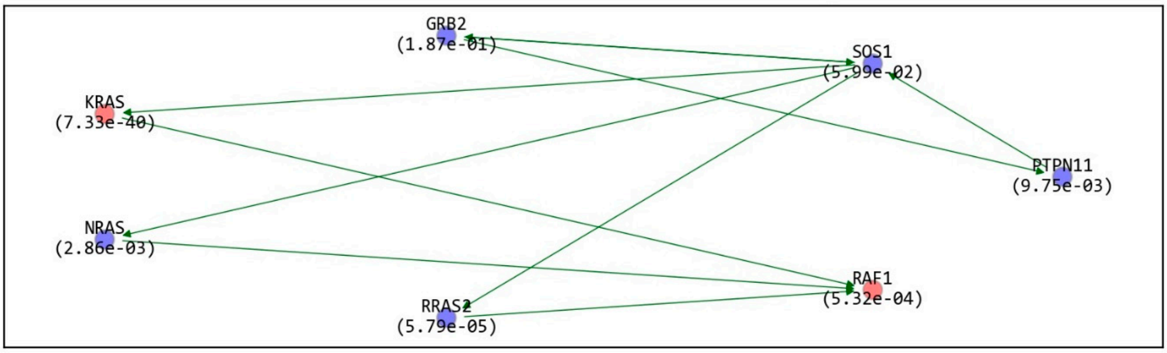

GIN2 for NRAS on KEGG in CRISPR screening

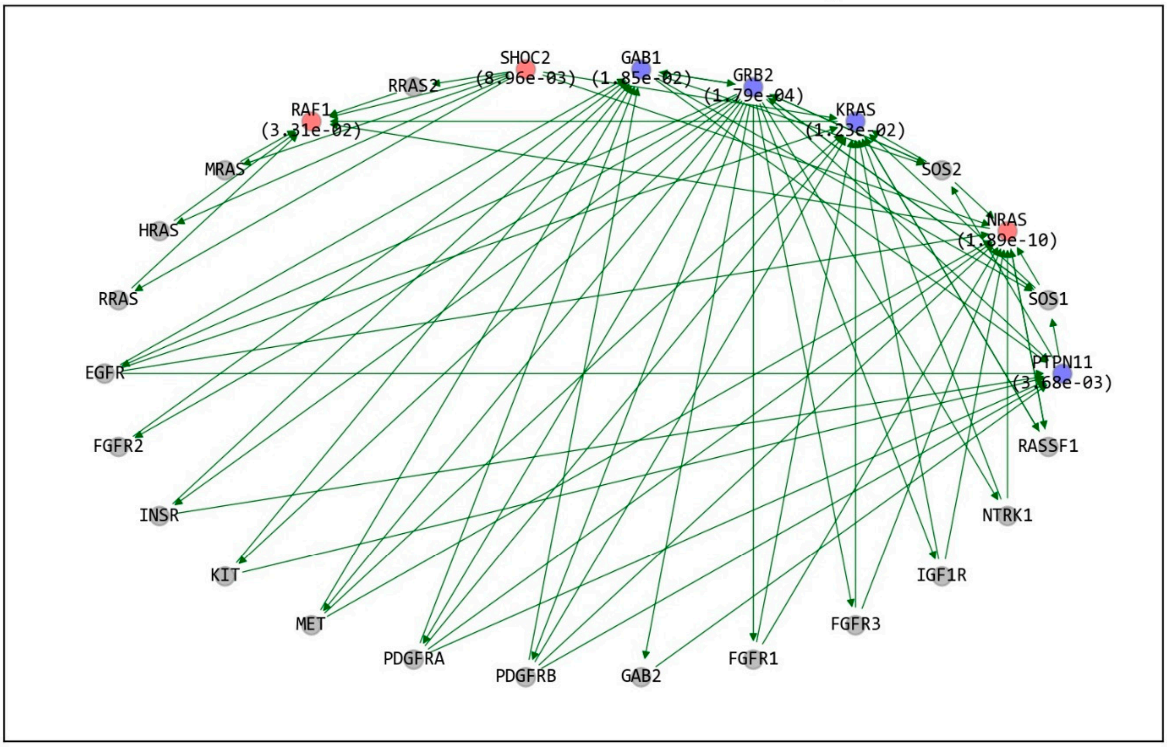

GIN1 for NRAS on KEGG in CRISPR screening

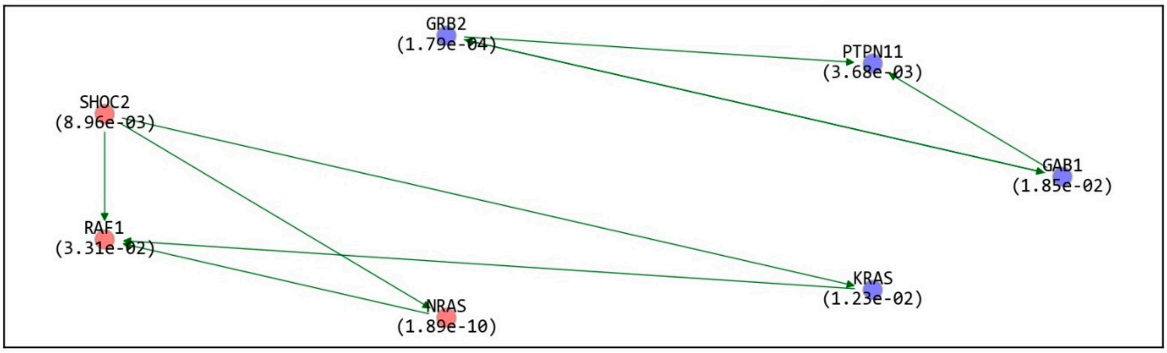

GIN2 for PIK3CA on KEGG in CRISPR screening

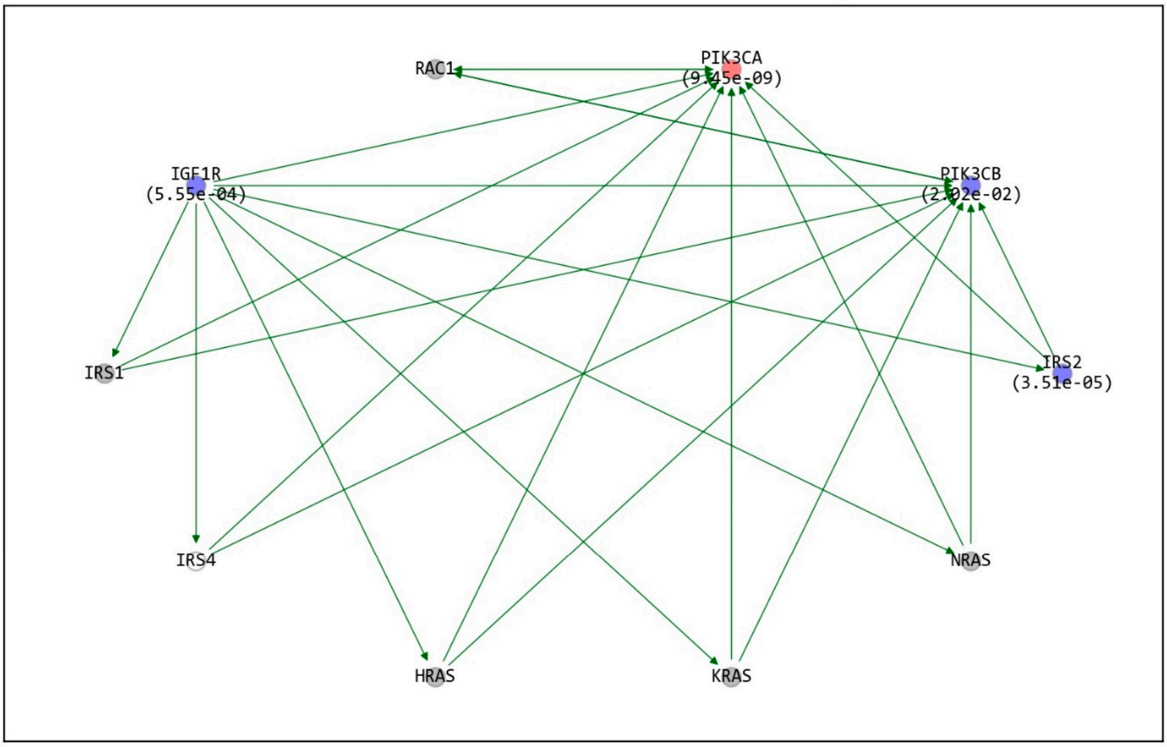

GIN1 for PIK3CA on KEGG in CRISPR screening

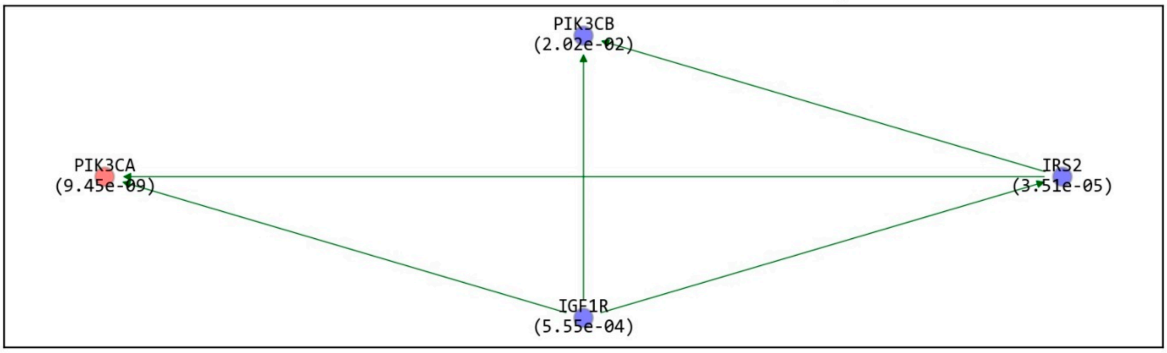

GIN2 for PTEN on KEGG in CRISPR screening

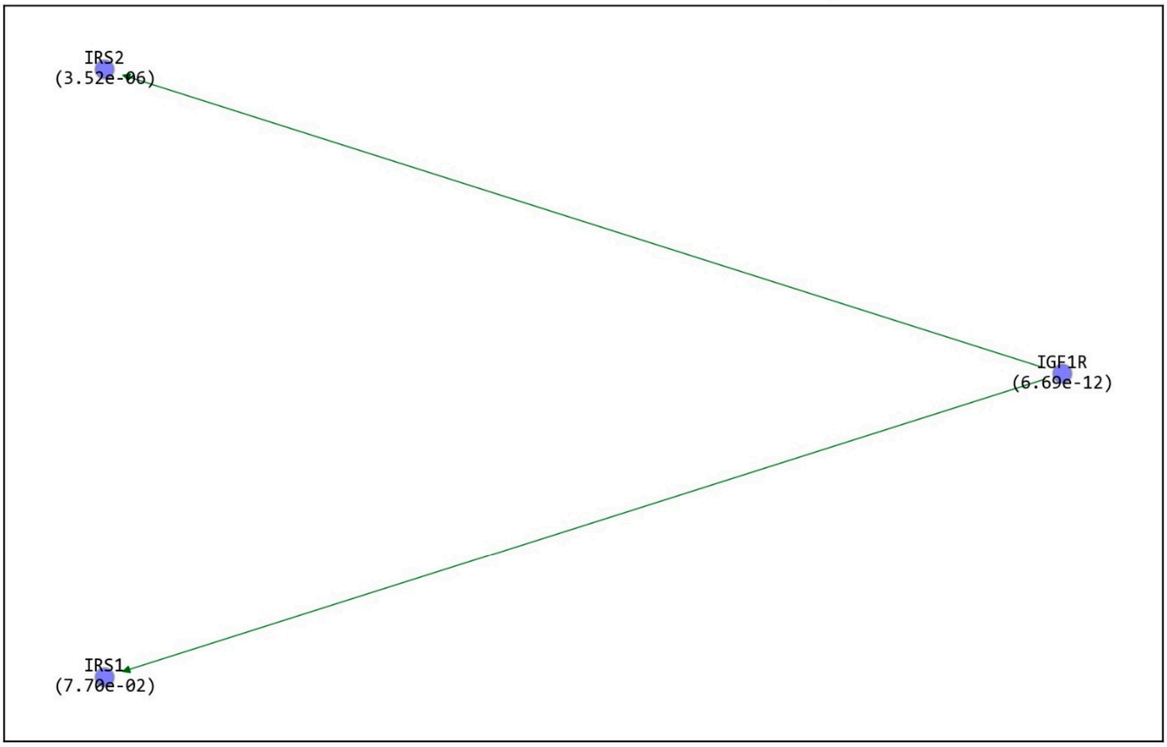

GIN1 for PTEN on KEGG in CRISPR screening

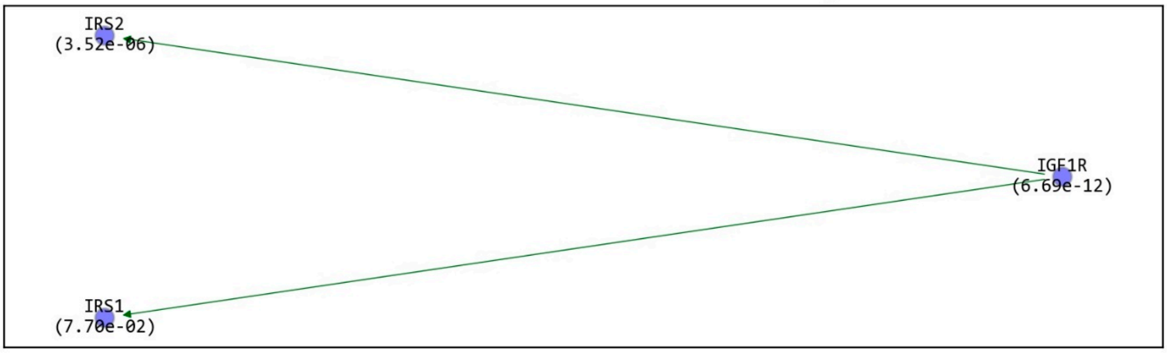

GIN2 for RB1 on KEGG in CRISPR screening

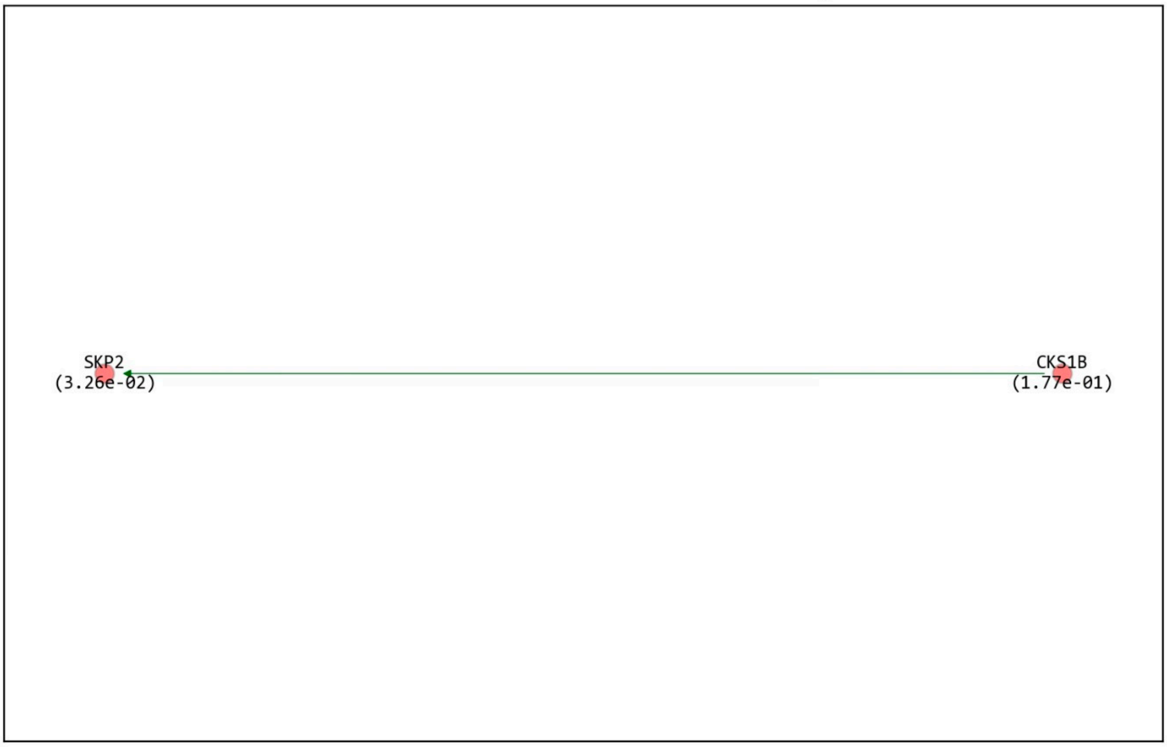

GIN1 for RB1 on KEGG in CRISPR screening

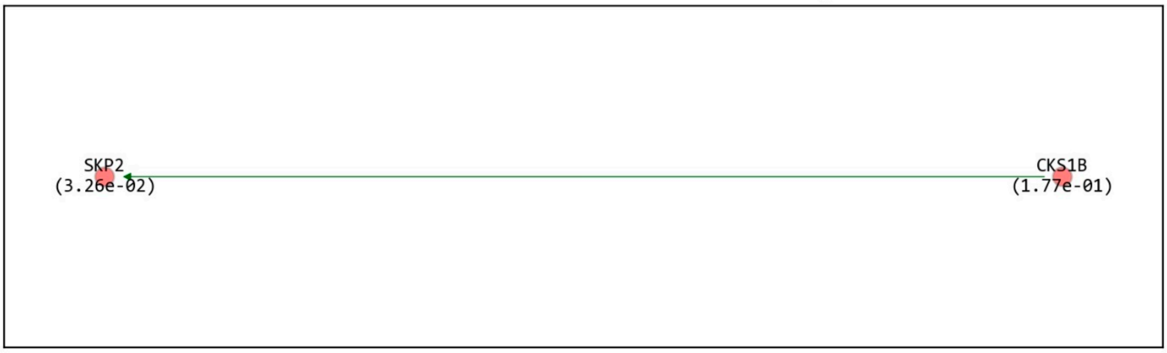

GIN2 for RPL22 on KEGG in CRISPR screening

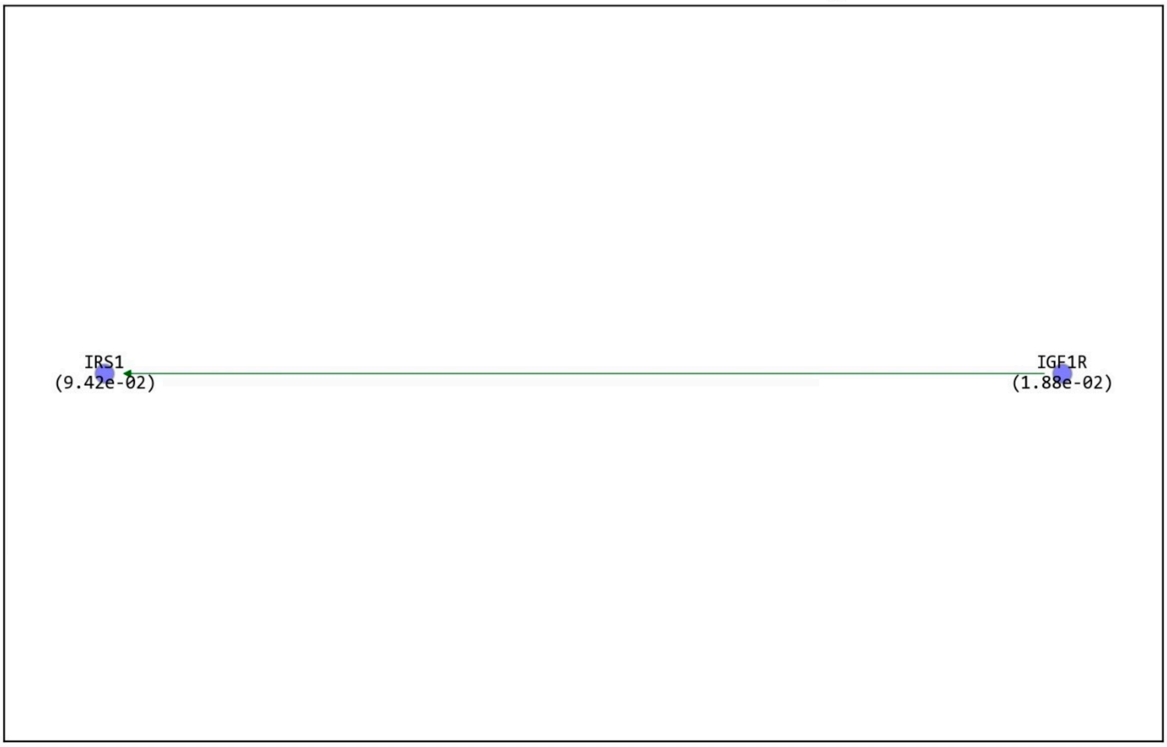

GIN1 for RPL22 on KEGG in CRISPR screening

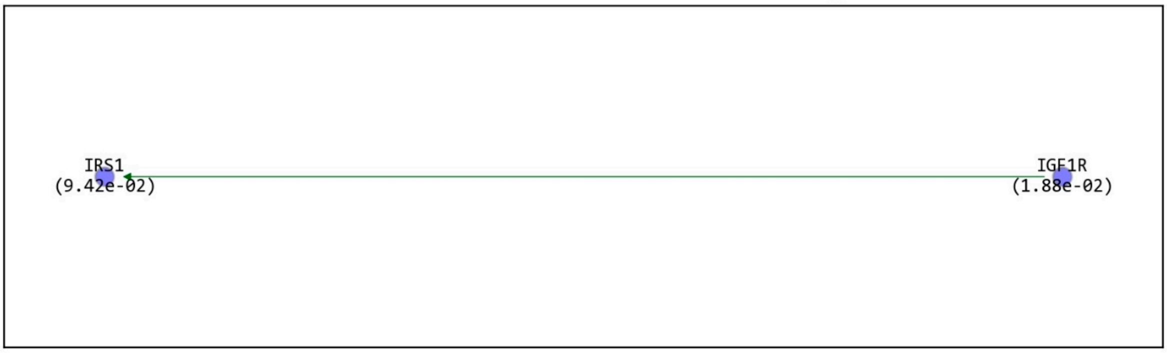

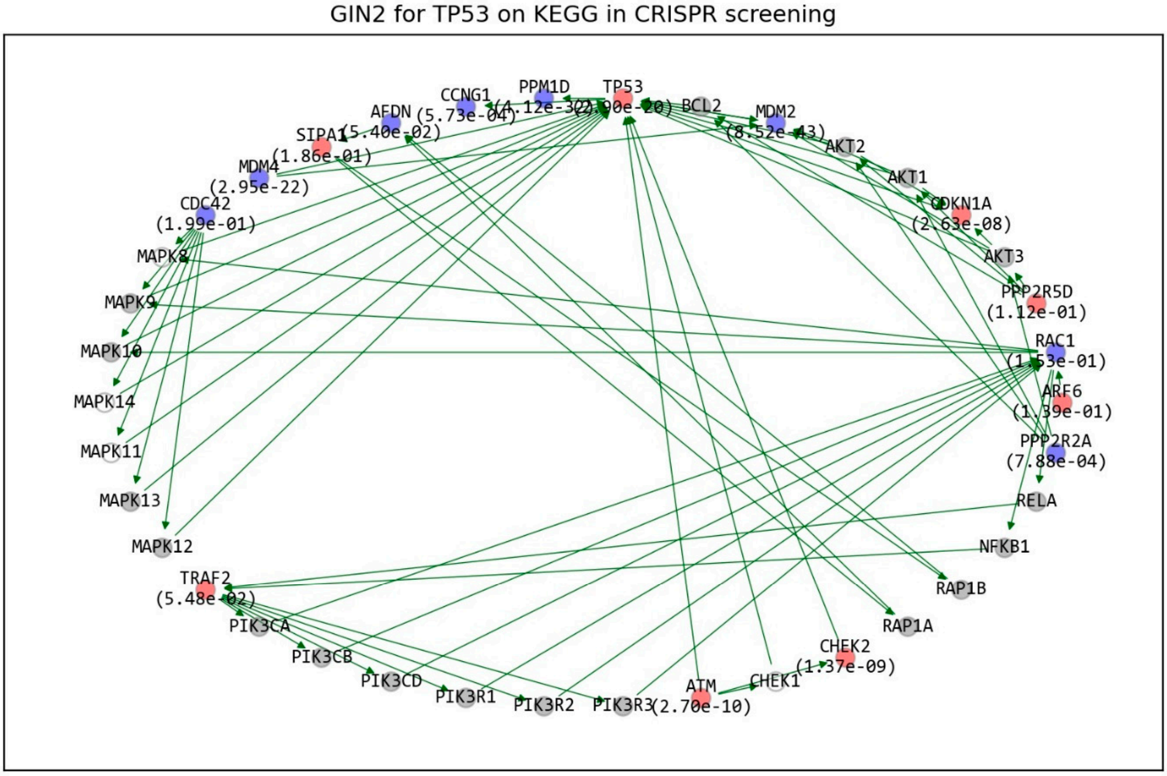
GIN2 for TP53 on KEGG in CRISPR screening
CCNG1
(5.73e-04)
PPM1D
(4.12e-32)
TP53
(2.90e-20)
BCL2
MDM2
(8.52e-43)
AFDN
(5.40e-02)
SIPA1
(1.86e-01)
MDM4
(2.95e-22)
AKT2
AKT1
CDKN1A
(2.63e-08)
CDC42
(1.99e-01)
AKT3
MAPK8
PPP2R5D
(1.12e-01)
MAPK9
MAPK10
RAC1
(1.53e-01)
ARF6
(1.39e-01)
MAPK14
MAPK11
PPP2R2A
(7.88e-04)
MAPK13
RELA
MAPK12
NFKB1
TRAF2
(5.48e-02)
RAP1B
PIK3CA
RAP1A
CHEK2
(1.37e-09)
PIK3CB
PIK3CD
CHEK1
ATM
(2.70e-10)
PIK3R1
PIK3R2
PIK3R3

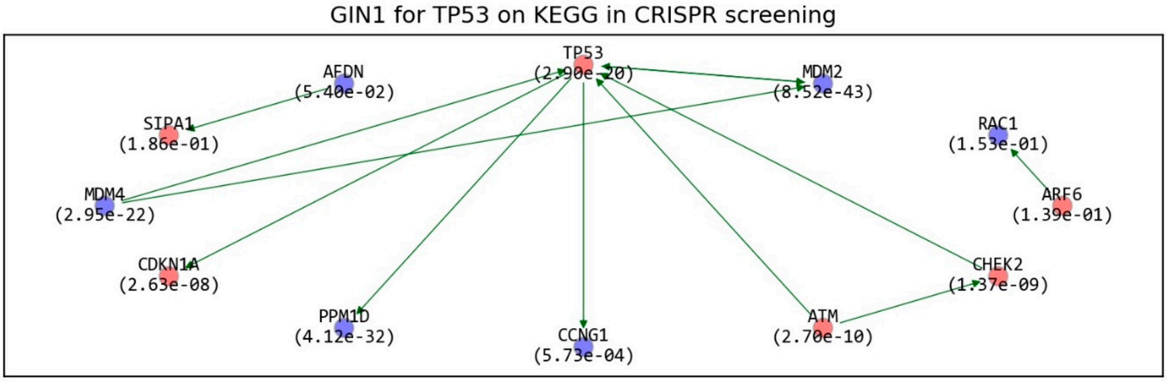
GIN1 for TP53 on KEGG in CRISPR screening
TP53
(2.90e-20)
AFDN
(5.40e-02)
MDM2
(8.52e-43)
SIPA1
(1.86e-01)
RAC1
(1.53e-01)
MDM4
(2.95e-22)
ARF6
(1.39e-01)
CDKN1A
(2.63e-08)
CHEK2
(1.37e-09)
PPM1D
(4.12e-32)
ATM
(2.70e-10)
CCNG1
(5.73e-04)

GIN2 for VHL on KEGG in CRISPR screening

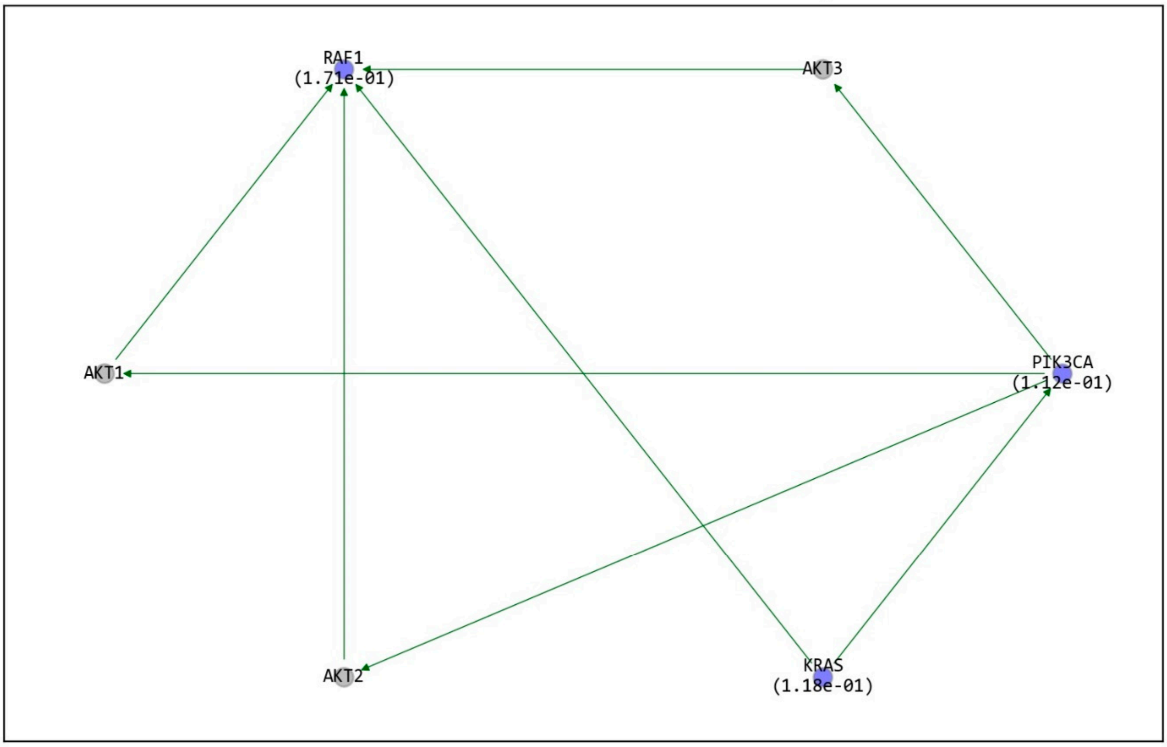

GIN1 for VHL on KEGG in CRISPR screening

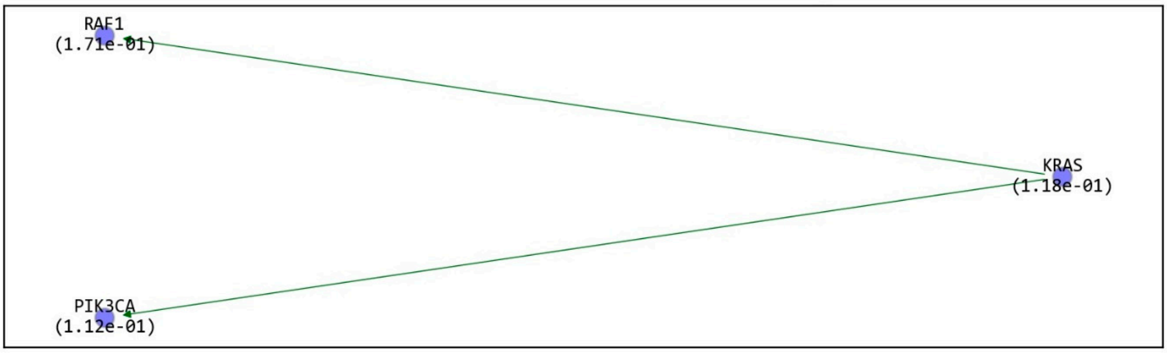

2. SPNs based on KEGG networks from shRNA screening

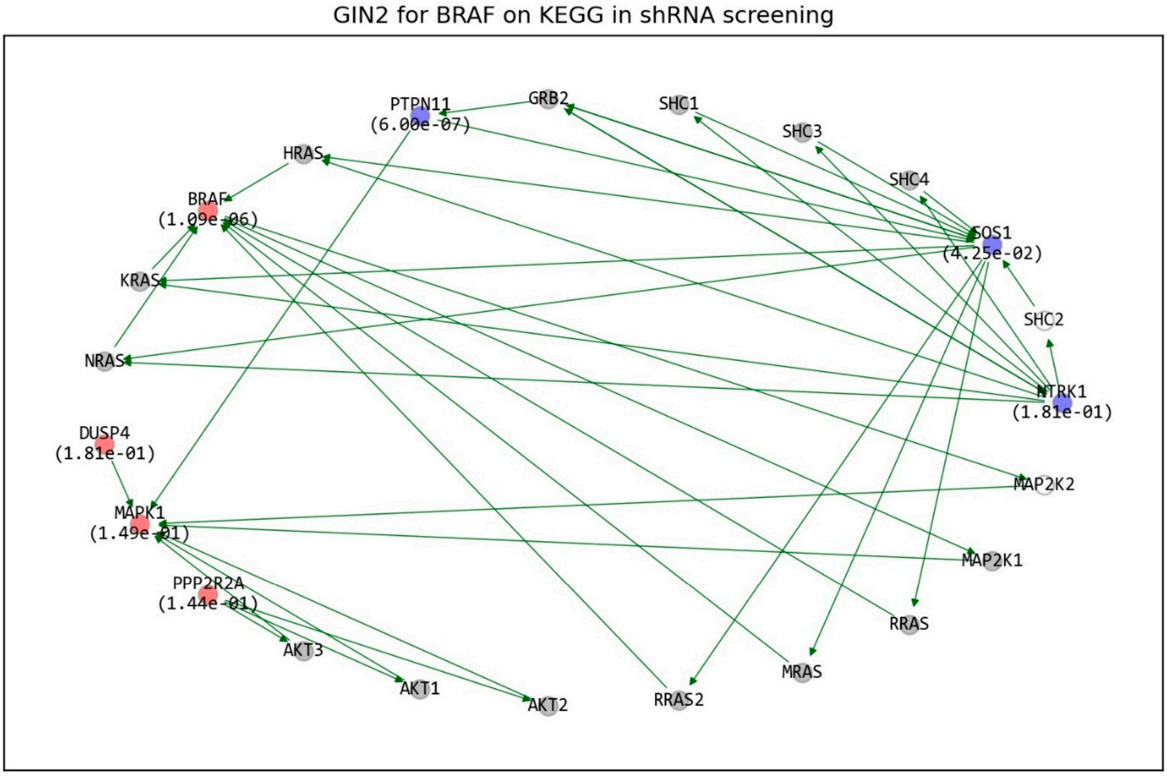

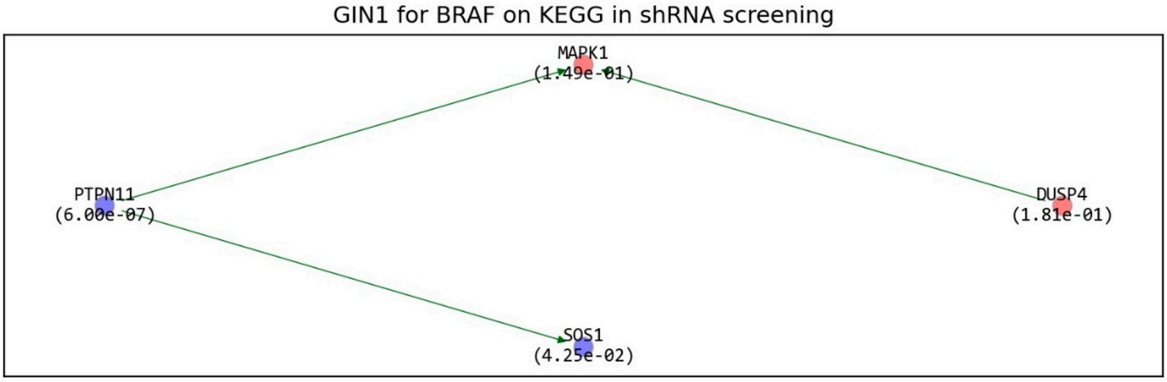

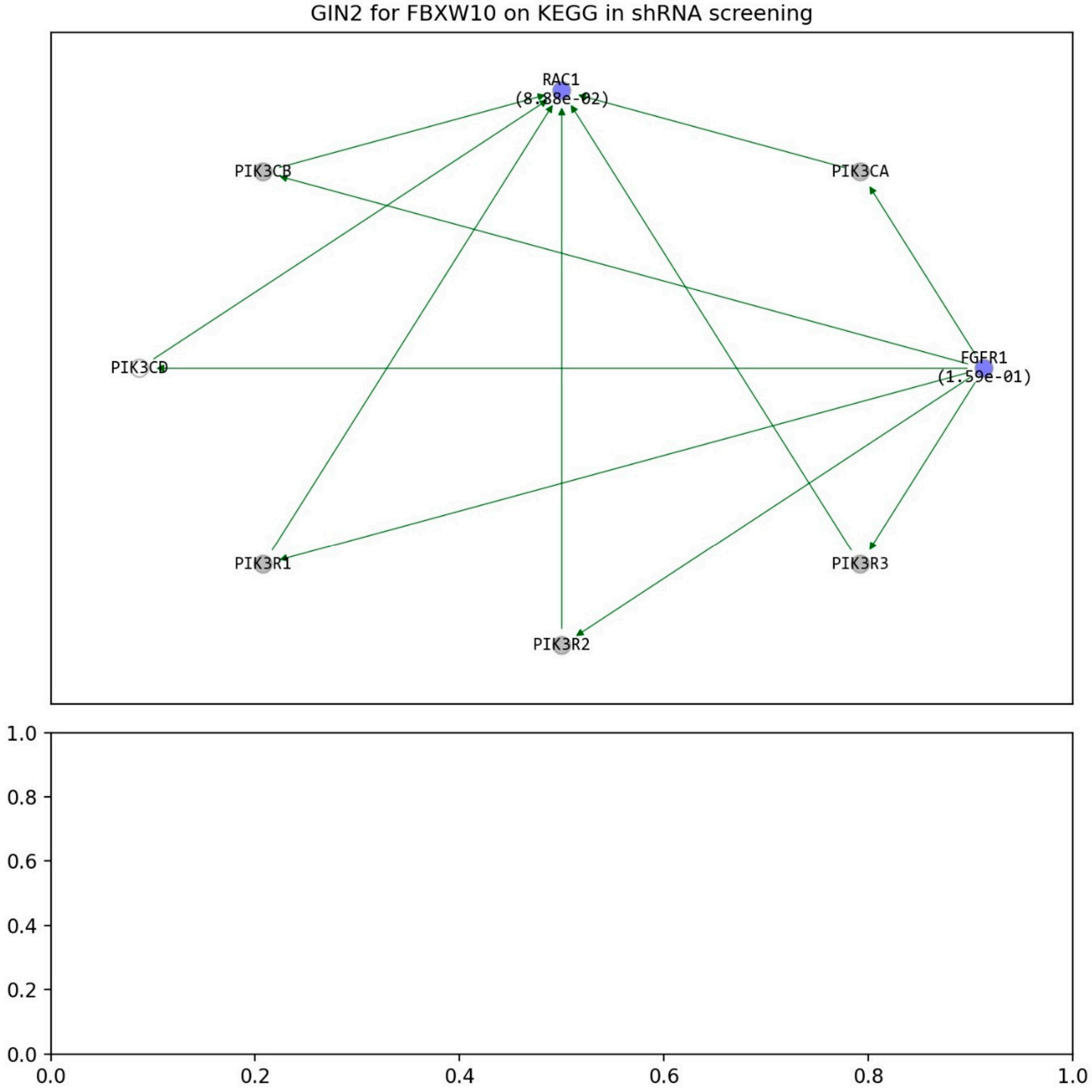
GIN2 for FBXW10 on KEGG in shRNA screening
RAC1
(8.88e-02)
PIK3CB
PIK3CA
PIK3CD
FGFR1
(1.59e-01)
PIK3R1
PIK3R3
PIK3R2
1.0
0.8
0.6
0.4
0.2
0.0
0.0
0.2
0.4
0.6
0.8
1.0

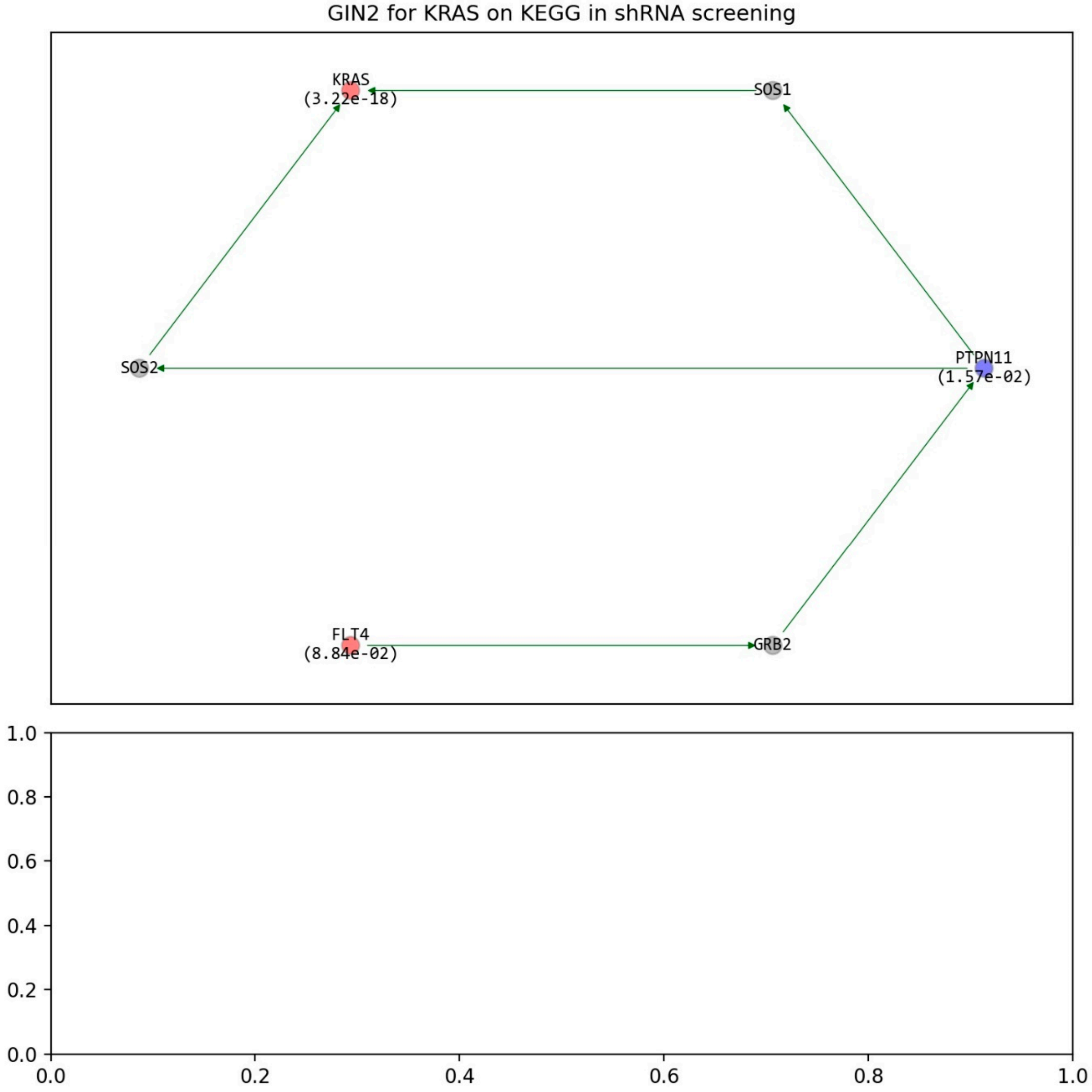
GIN2 for KRAS on KEGG in shRNA screening
KRAS
(3.22e-18)
SOS1
SOS2
PTPN11
(1.57e-02)
FLT4
(8.84e-02)
GRB2
1.0
0.8
0.6
0.4
0.2
0.0
0.0
0.2
0.4
0.6
0.8
1.0

GIN2 for PIK3CA on KEGG in shRNA screening

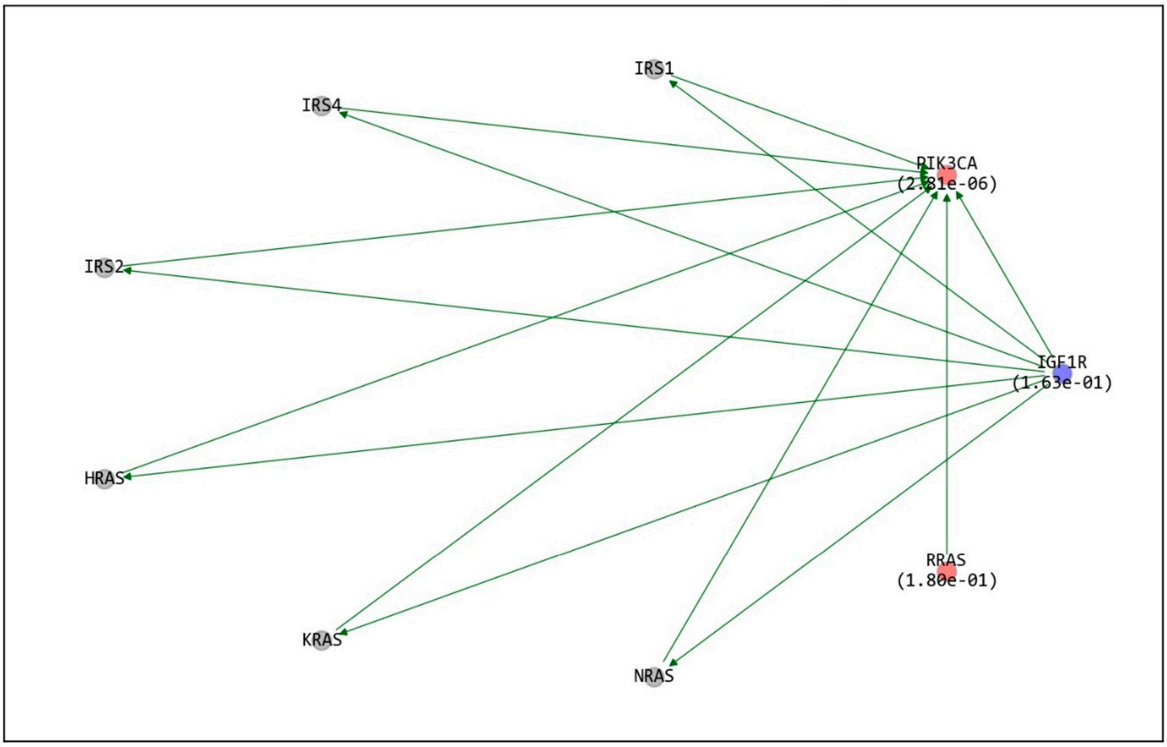

GIN1 for PIK3CA on KEGG in shRNA screening

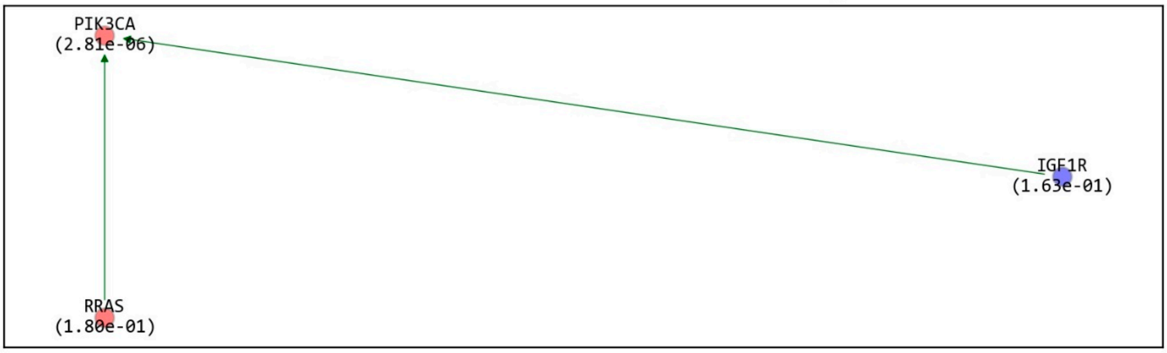

GIN2 for PTEN on KEGG in shRNA screening

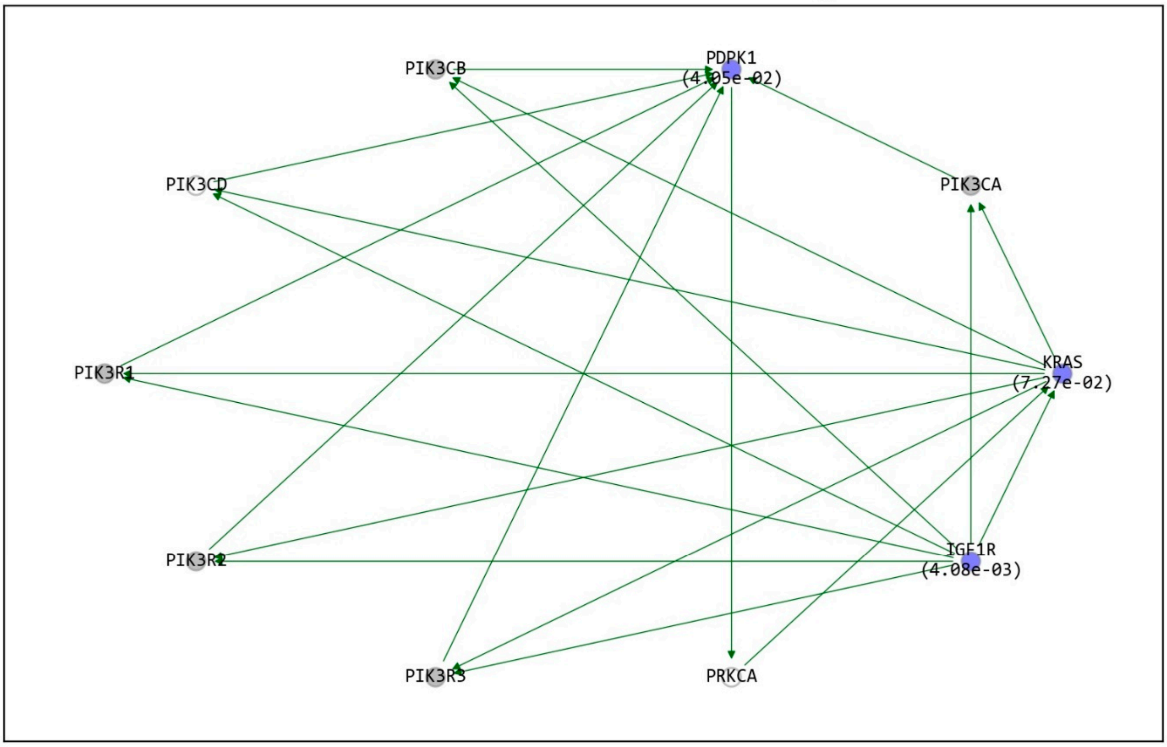

GIN1 for PTEN on KEGG in shRNA screening

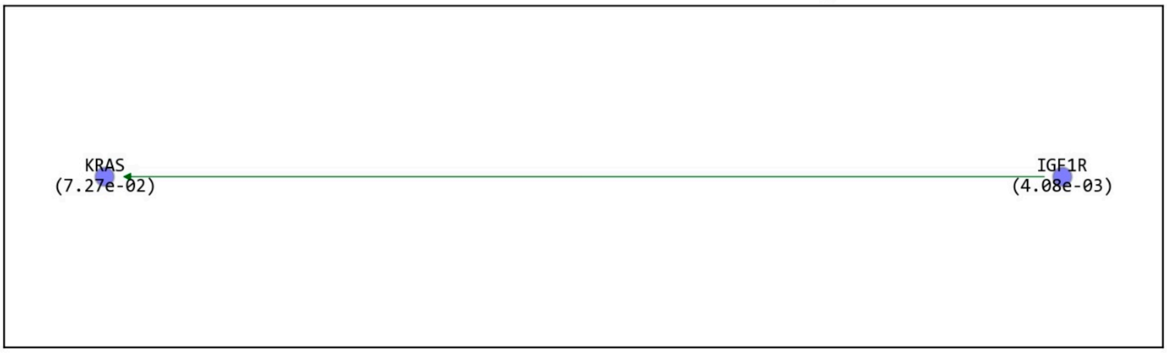

GIN2 for RB1 on KEGG in shRNA screening

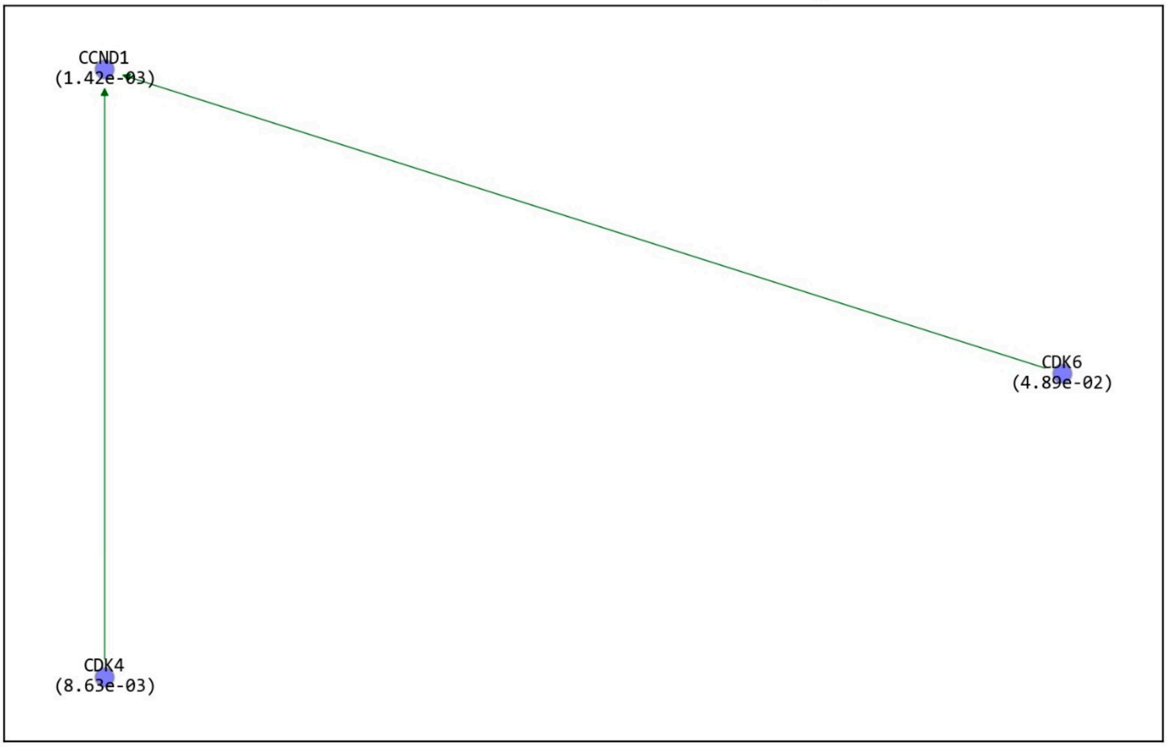

GIN1 for RB1 on KEGG in shRNA screening

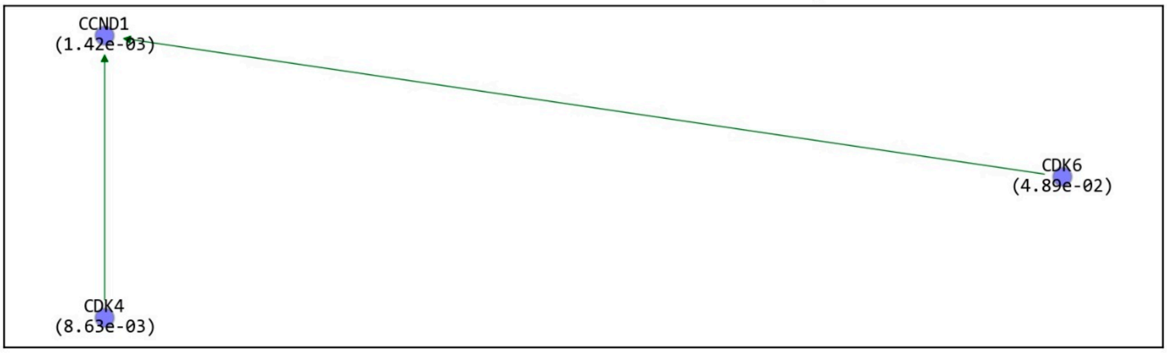

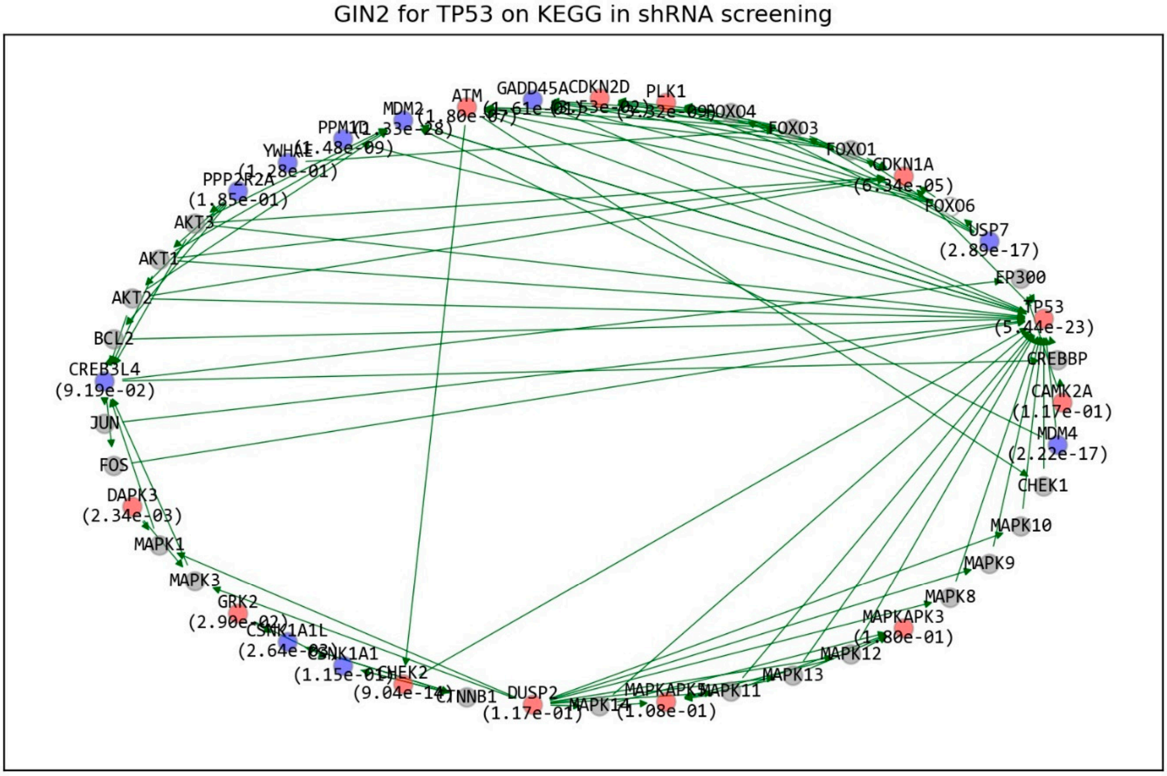
GIN2 for TP53 on KEGG in shRNA screening
ATM
GADD45A
CDKN2D
PLK1
FOXO4
MDM2
PPM1D
(1.48e-09)
FOXO3
YWHAE
(1.28e-01)
FOXO1
CDKN1A
(6.34e-05)
PPP2R2A
(1.85e-01)
FOXO6
AKT3
USP7
(2.89e-17)
AKT1
EP300
AKT2
TP53
BCL2
CREBBP
CREB3L4
(9.19e-02)
CAMK2A
(1.17e-01)
JUN
MDM4
(2.22e-17)
FOS
CHEK1
DAPK3
(2.34e-03)
MAPK10
MAPK1
MAPK9
MAPK3
MAPK8
GRK2
MAPKAPK3
CSNK1A1L
MAPK12
CSNK1A1
MAPK13
CHEK2
MAPKAPK5
MAPK11
CTNNB1
DUSP2
(1.17e-01)
MAPK14
(1.08e-01)

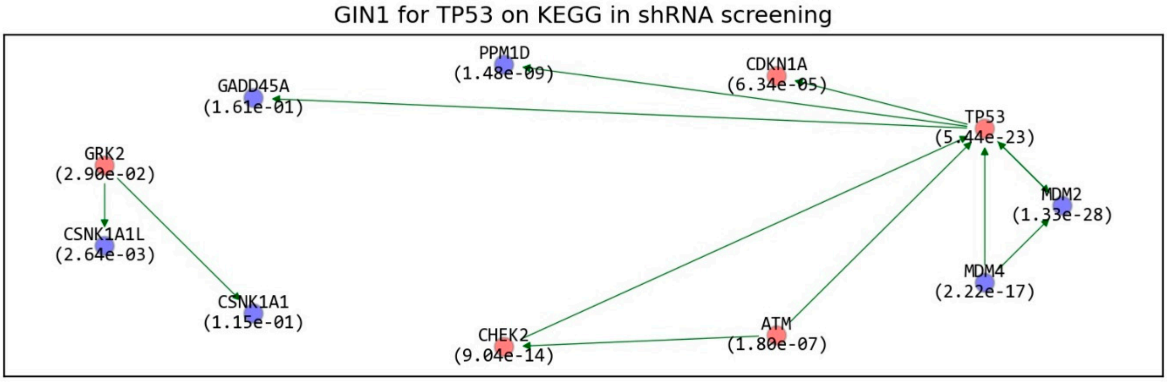
GIN1 for TP53 on KEGG in shRNA screening
PPM1D
(1.48e-09)
CDKN1A
(6.34e-05)
GADD45A
(1.61e-01)
TP53
(5.44e-23)
GRK2
(2.90e-02)
MDM2
(1.33e-28)
CSNK1A1L
(2.64e-03)
MDM4
(2.22e-17)
CSNK1A1
(1.15e-01)
ATM
(1.80e-07)
CHEK2
(9.04e-14)

Figure S6. SPNs on PPI networks from (a) CRISPR and (b) shRNA screenings for the mutated genes in Figure 5.

1. SPNs based on PPI networks from CRSPR screening

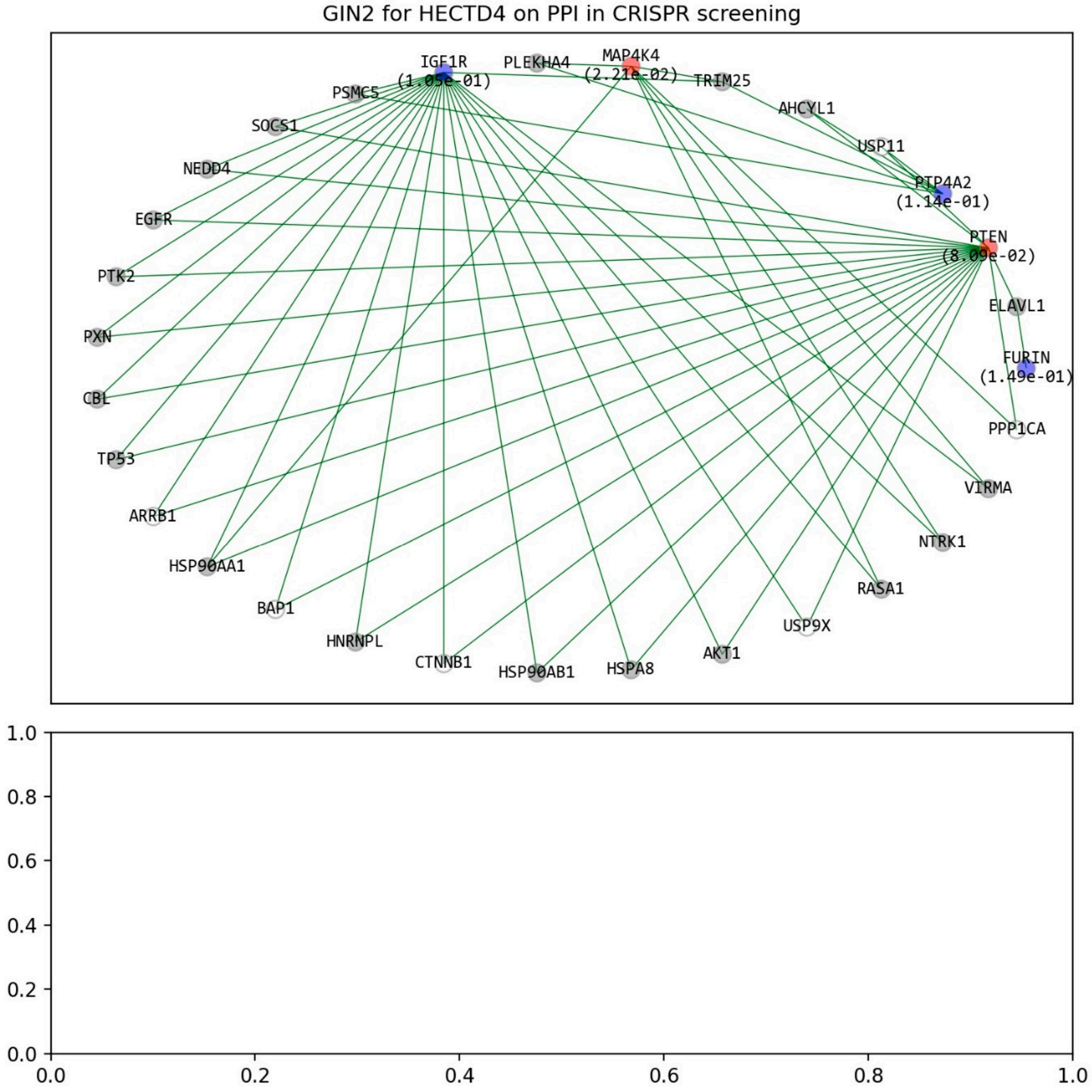

GIN2 for MAGEA2 on PPI in CRISPR screening

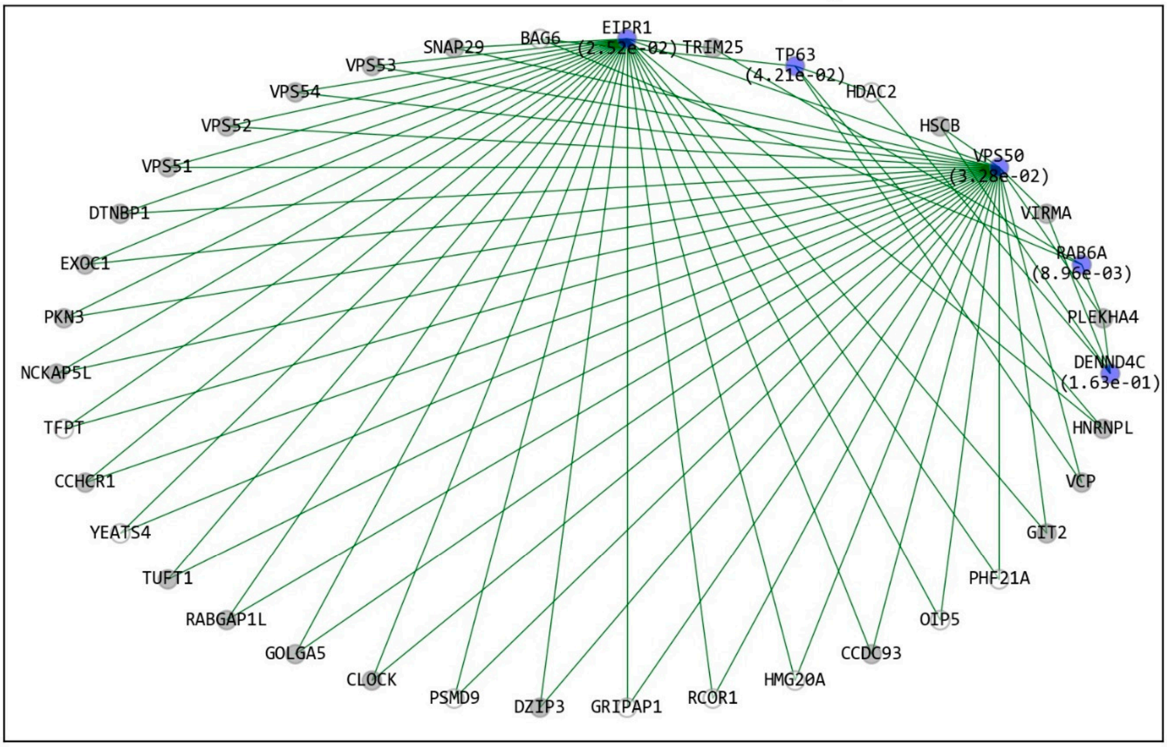

GIN1 for MAGEA2 on PPI in CRISPR screening

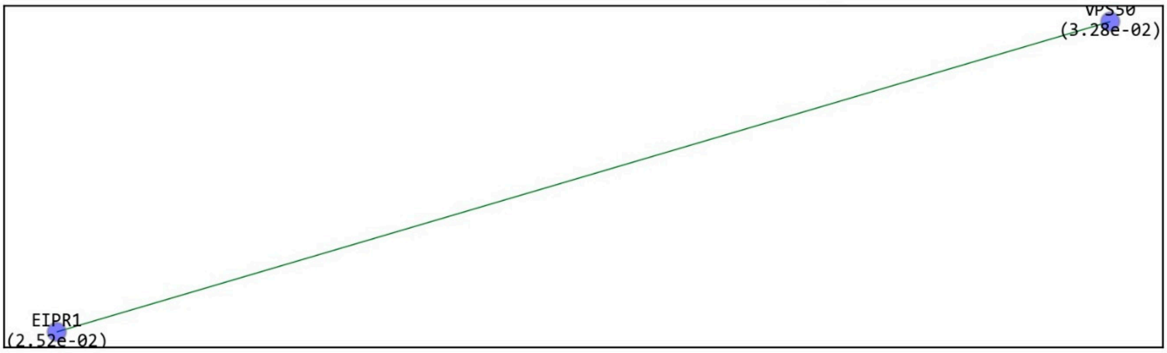

GIN2 for CCDC57 on PPI in CRISPR screening

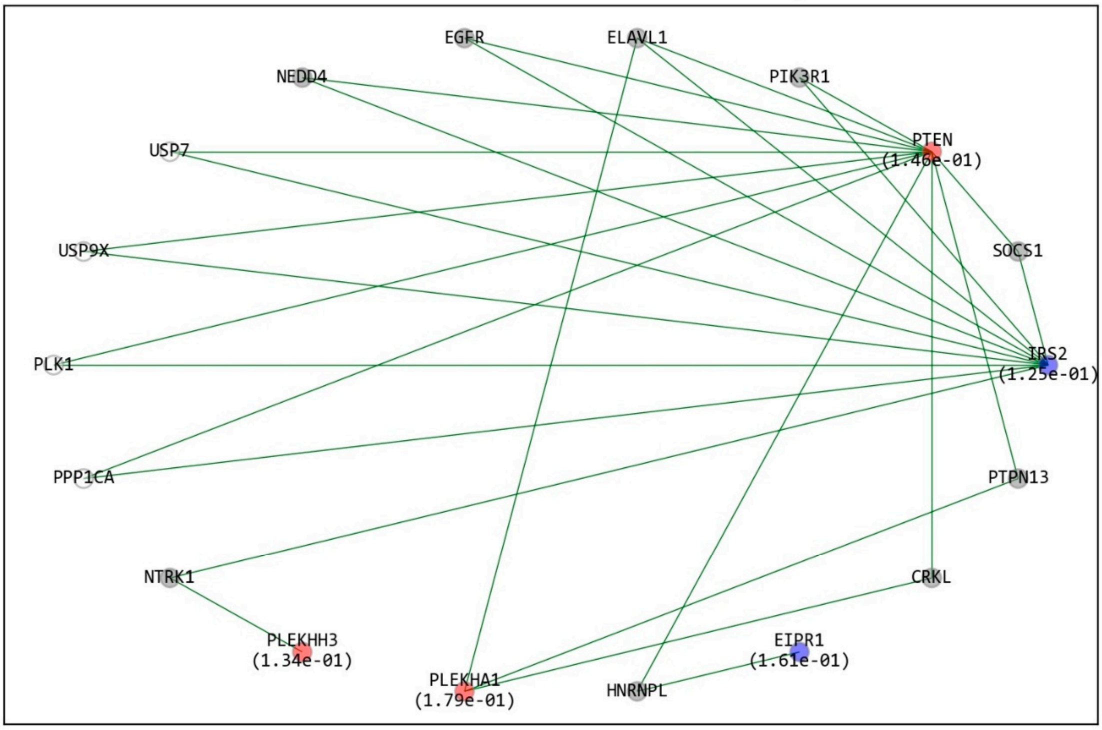

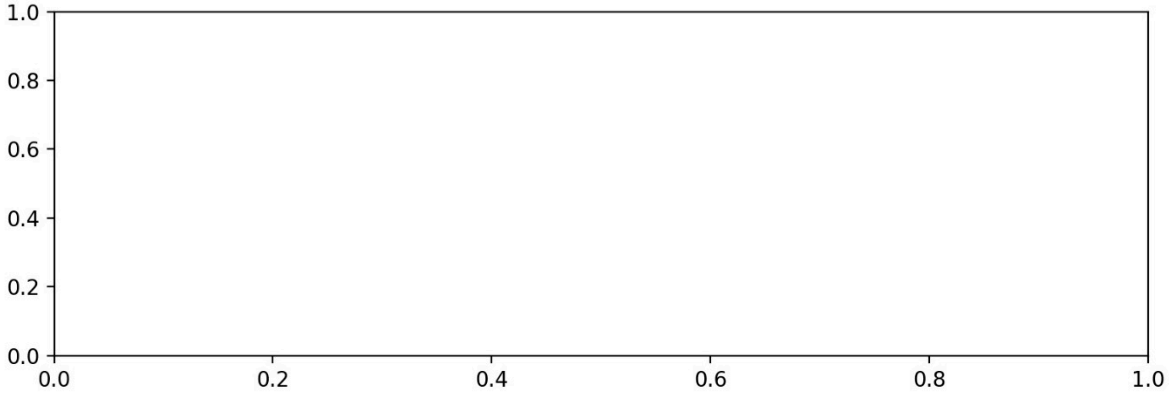

GIN2 for VHL on PPI in CRISPR screening

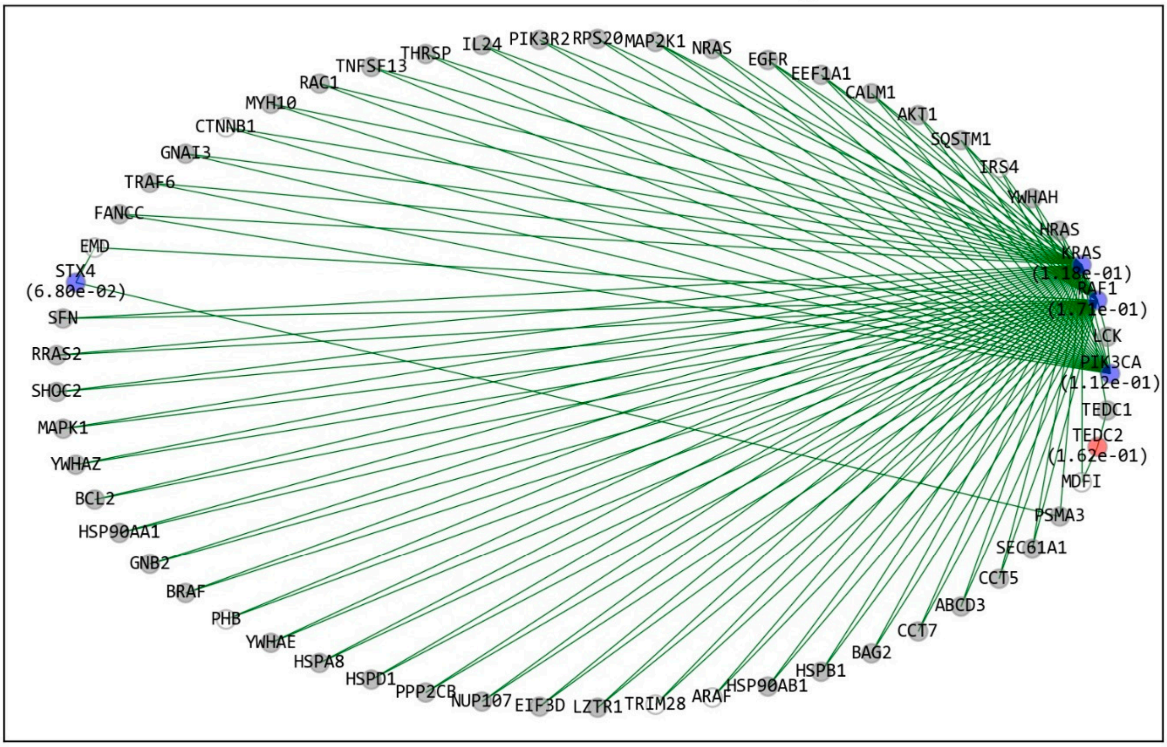

GIN1 for VHL on PPI in CRISPR screening

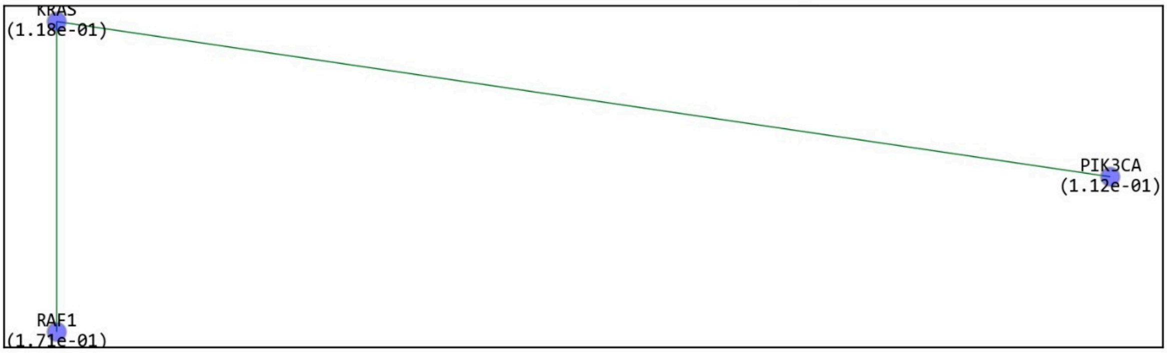

GIN2 for PIK3CA on PPI in CRISPR screening

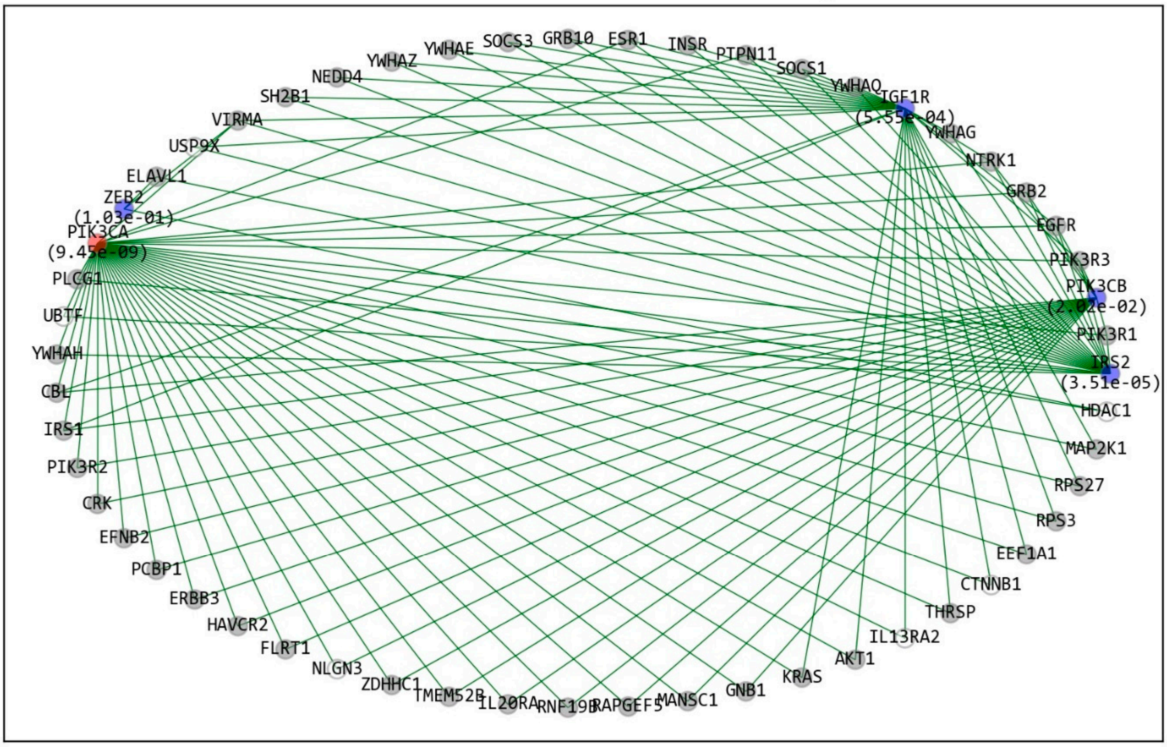

GIN1 for PIK3CA on PPI in CRISPR screening

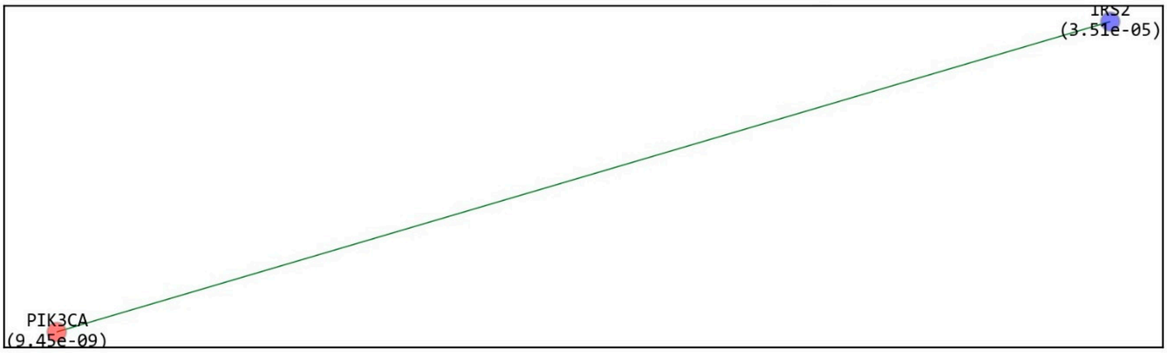

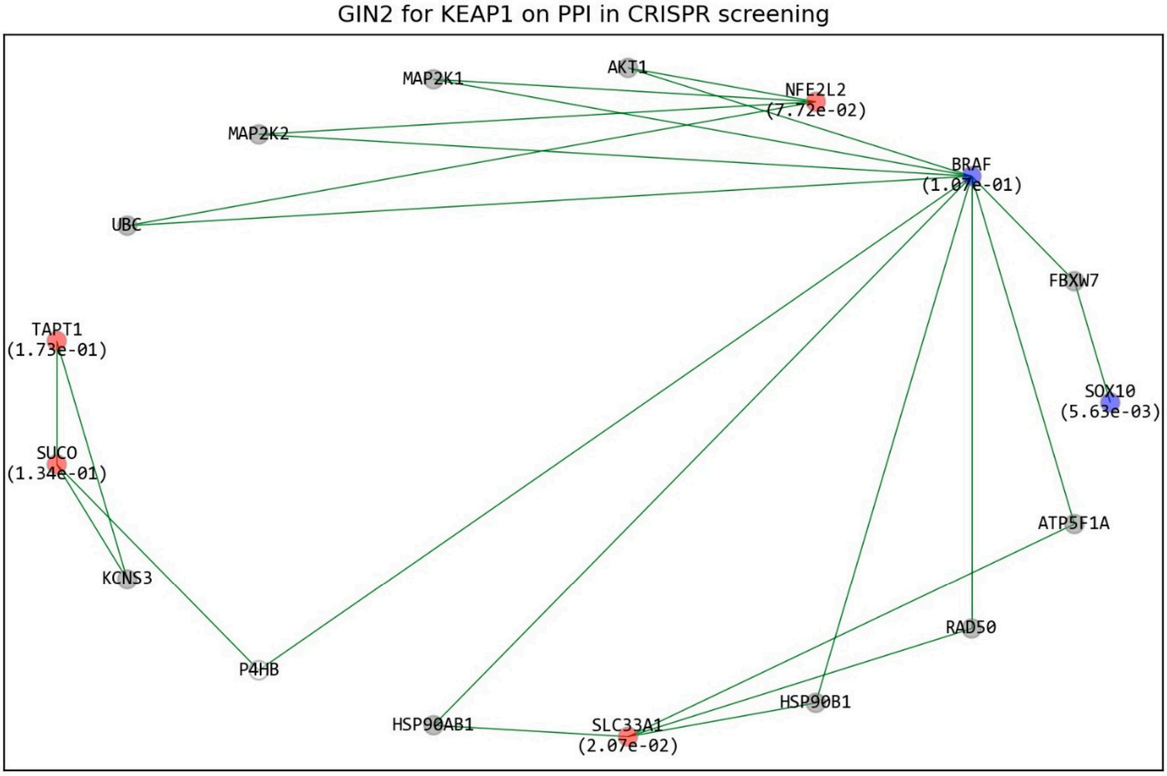
GIN2 for KEAP1 on PPI in CRISPR screening
MAP2K1
AKT1
NFE2L2
(7.72e-02)
MAP2K2
BRAF
(1.07e-01)
UBC
FBXW7
TAPT1
(1.73e-01)
SOX10
(5.63e-03)
SUCO
(1.34e-01)
ATP5F1A
KCNS3
RAD50
P4HB
HSP90B1
HSP90AB1
SLC33A1
(2.07e-02)

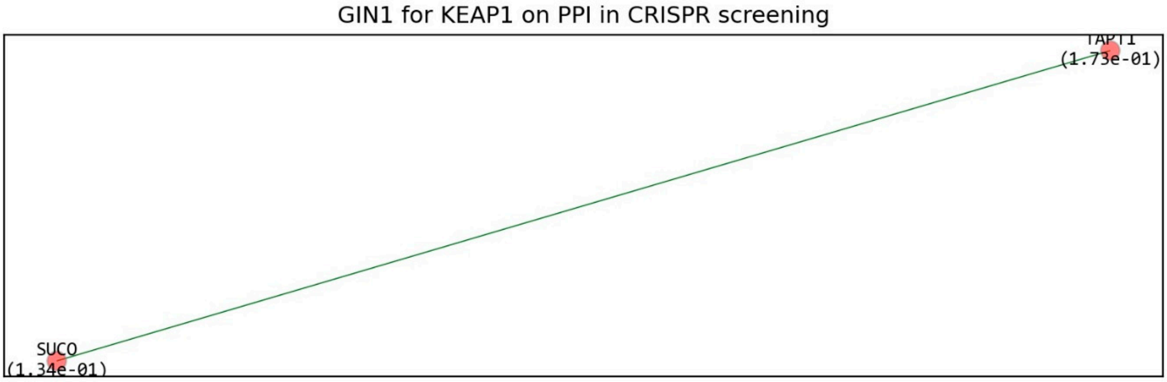
GIN1 for KEAP1 on PPI in CRISPR screening
TAPT1
(1.73e-01)
SUCO
(1.34e-01)

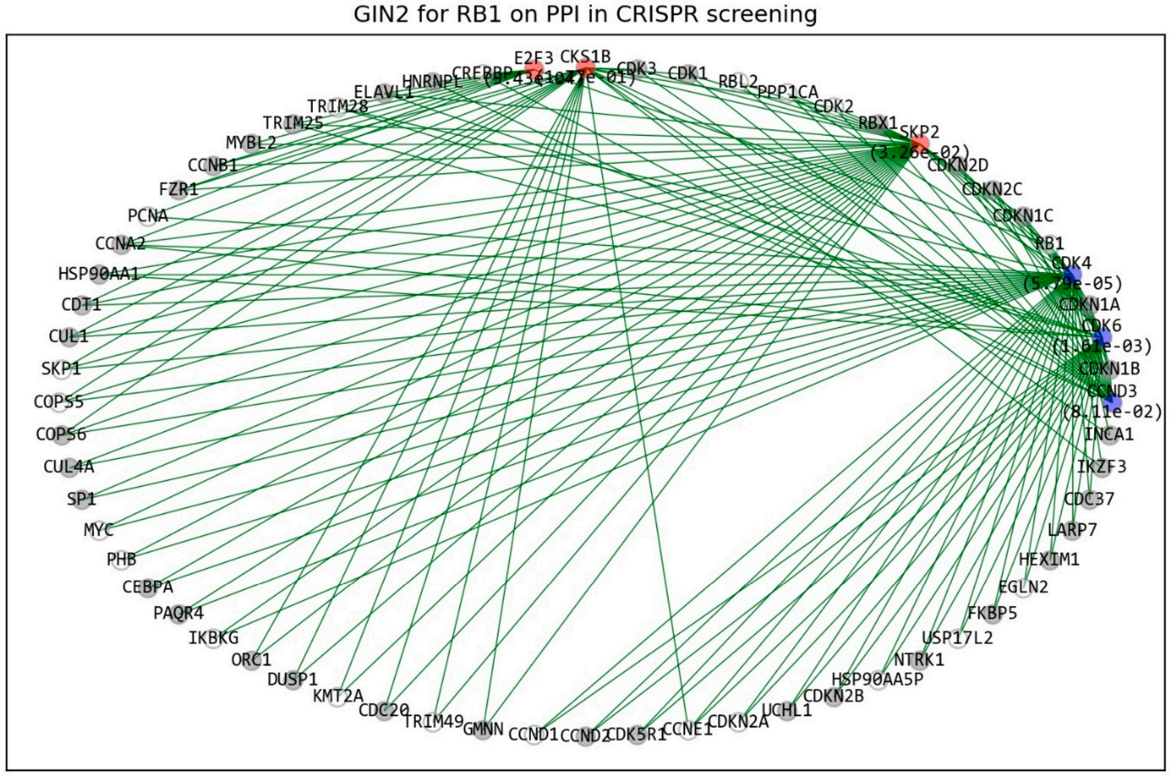
GIN2 for RB1 on PPI in CRISPR screening
E2F3
CKS1B
CDK3
CDK1
RBL2
PPP1CA
CDK2
RBX1
SKP2
(3.26e-02)
CDKN2D
CDKN2C
CDKN1C
RB1
CDK4
(5.79e-05)
CDKN1A
CDK6
(1.61e-03)
CDKN1B
CCND3
(8.11e-02)
INCA1
IKZF3
CDC37
LARP7
HEXIM1
EGLN2
FKBP5
USP17L2
NTRK1
HSP90AA5P
CDKN2B
UCHL1
CDKN2A
CCNE1
CDK5R1
CCND2
CCND1
GMNN
TRIM49
CDC20
KMT2A
DUSP1
ORC1
IKBKG
PAQR4
CEBPA
PHB
MYC
SP1
CUL4A
COPS6
COPS5
SKP1
CUL1
CDT1
HSP90AA1
CCNA2
PCNA
FZR1
CCNB1
MYBL2
TRIM25
TRIM28
ELAVL1
HNRNPL

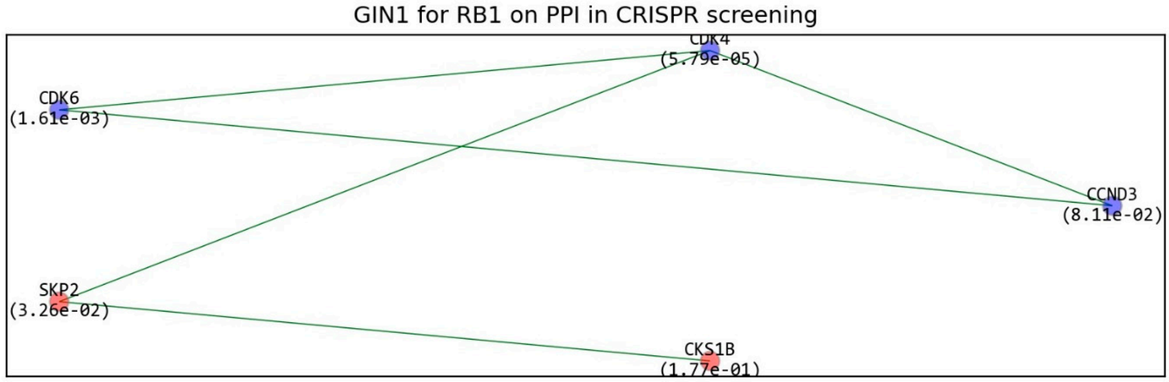
GIN1 for RB1 on PPI in CRISPR screening
CDK4
(5.79e-05)
CDK6
(1.61e-03)
CCND3
(8.11e-02)
SKP2
(3.26e-02)
CKS1B
(1.77e-01)

GIN1 for NRAS on PPI in CRISPR screening

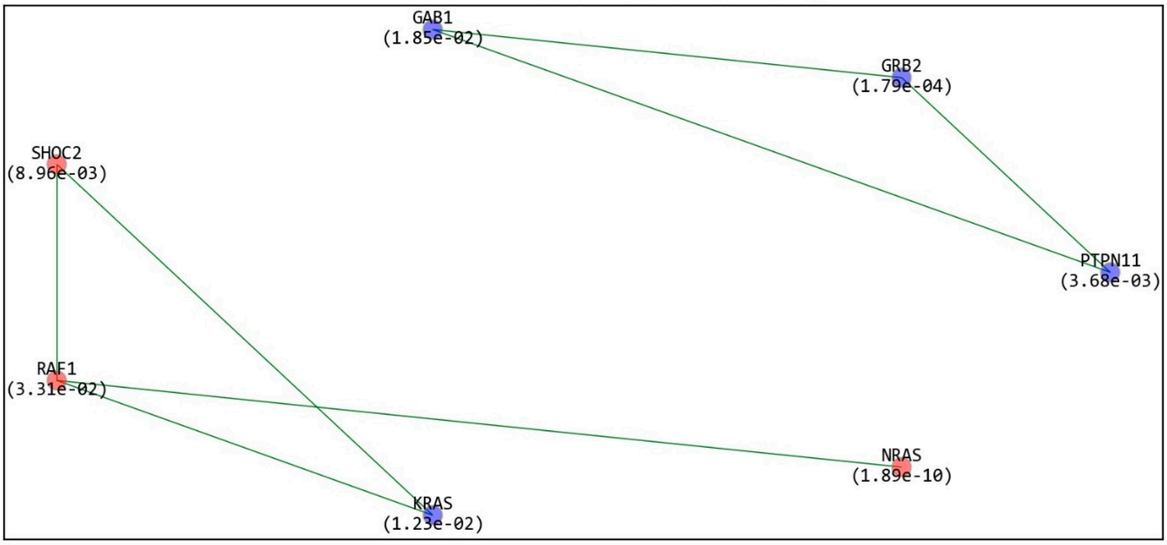

GIN1 for CTCF on PPI in CRISPR screening

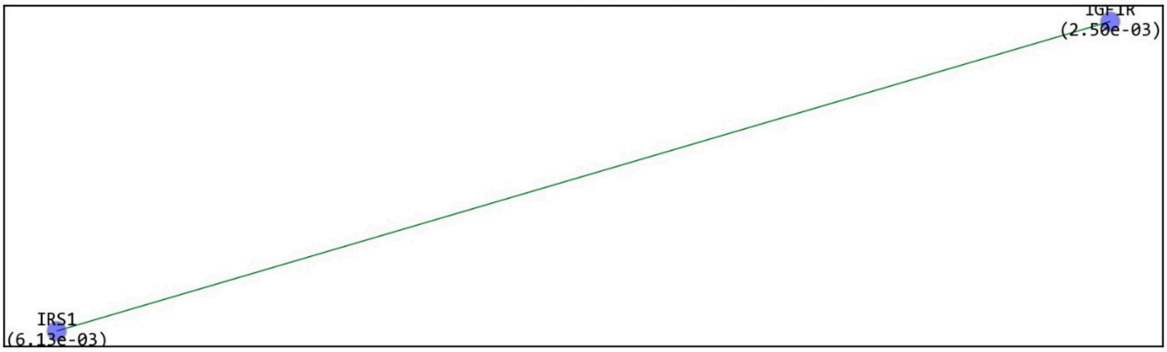

GIN2 for RPL22 on PPI in CRISPR screening

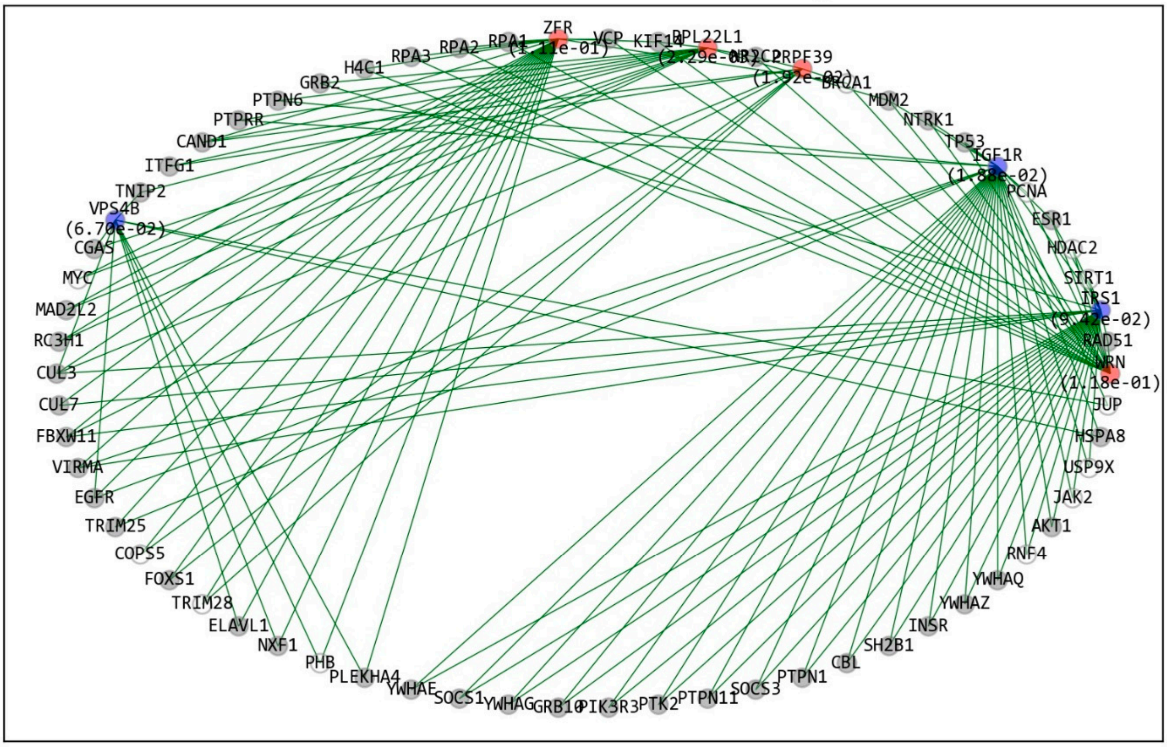

GIN1 for RPL22 on PPI in CRISPR screening

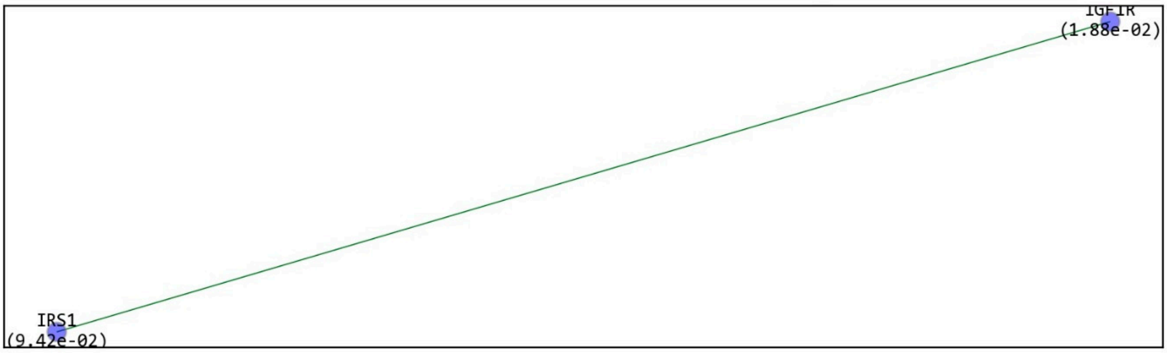

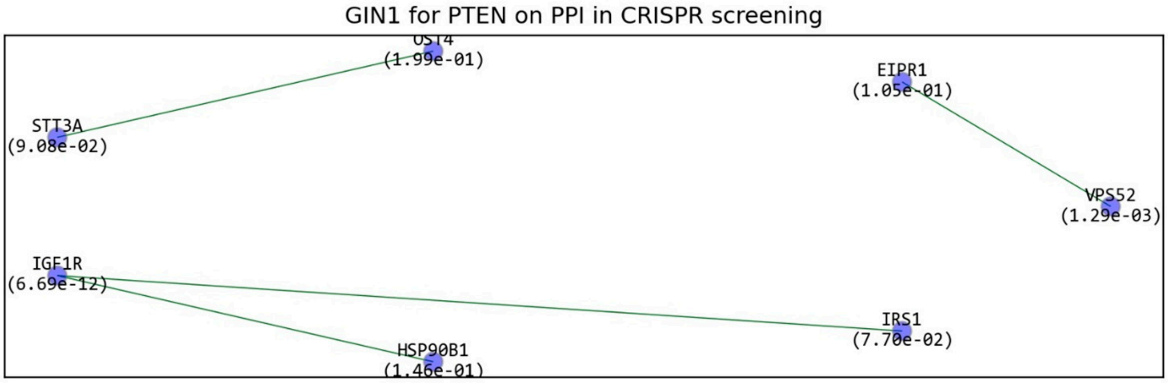
GIN1 for PTEN on PPI in CRISPR screening
OST4
(1.99e-01)
EIPR1
(1.05e-01)
STT3A
(9.08e-02)
VPS52
(1.29e-03)
IGF1R
(6.69e-12)
IRS1
(7.70e-02)
HSP90B1
(1.46e-01)

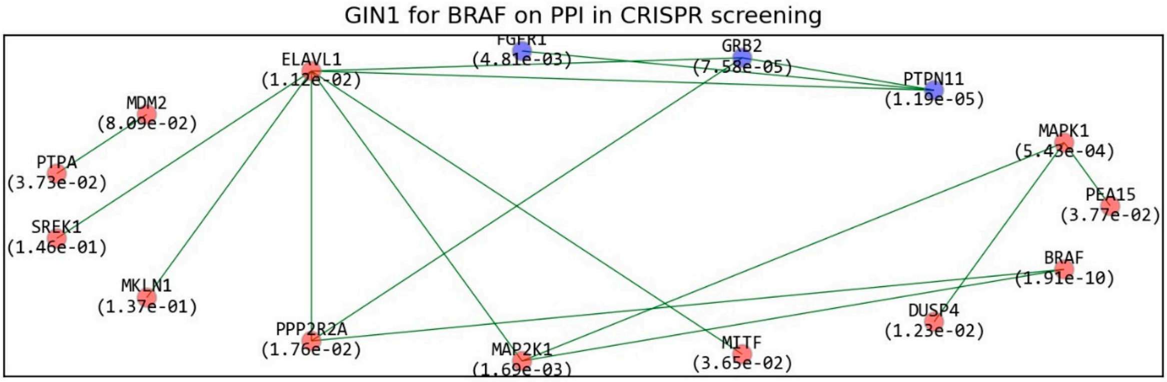
GIN1 for BRAF on PPI in CRISPR screening
FGFR1
(4.81e-03)
ELAVL1
(1.12e-02)
GRB2
(7.58e-05)
PTPN11
(1.19e-05)
MDM2
(8.09e-02)
PTPA
(3.73e-02)
MAPK1
(5.43e-04)
PEA15
(3.77e-02)
SREK1
(1.46e-01)
MKLN1
(1.37e-01)
BRAF
(1.91e-10)
DUSP4
(1.23e-02)
PPP2R2A
(1.76e-02)
MAP2K1
(1.69e-03)
MITF
(3.65e-02)

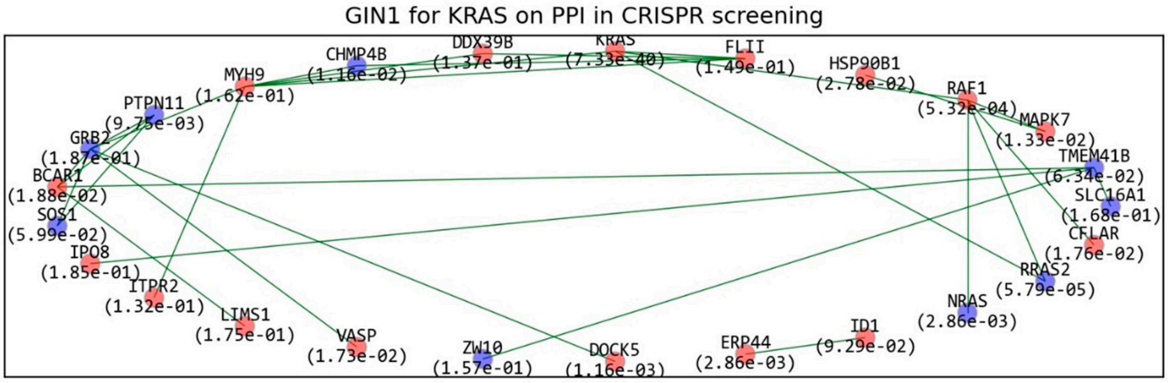
GIN1 for KRAS on PPI in CRISPR screening
KRAS
(7.33e-40)
DDX39B
(1.37e-01)
CHMP4B
(1.16e-02)
FLII
(1.49e-01)
HSP90B1
(2.78e-02)
MYH9
(1.62e-01)
RAF1
(5.32e-04)
PTPN11
(9.75e-03)
MAPK7
(1.33e-02)
GRB2
(1.87e-01)
TMEM41B
(6.34e-02)
BCAR1
(1.88e-02)
SLC16A1
(1.68e-01)
SOS1
(5.99e-02)
CFLAR
(1.76e-02)
IPO8
(1.85e-01)
RRAS2
(5.79e-05)
ITPR2
(1.32e-01)
NRAS
(2.86e-03)
LIMS1
(1.75e-01)
ID1
(9.29e-02)
VASP
(1.73e-02)
ERP44
(2.86e-03)
ZW10
(1.57e-01)
DOCK5
(1.16e-03)

2. SPNs based on PPI networks from shRNA screening

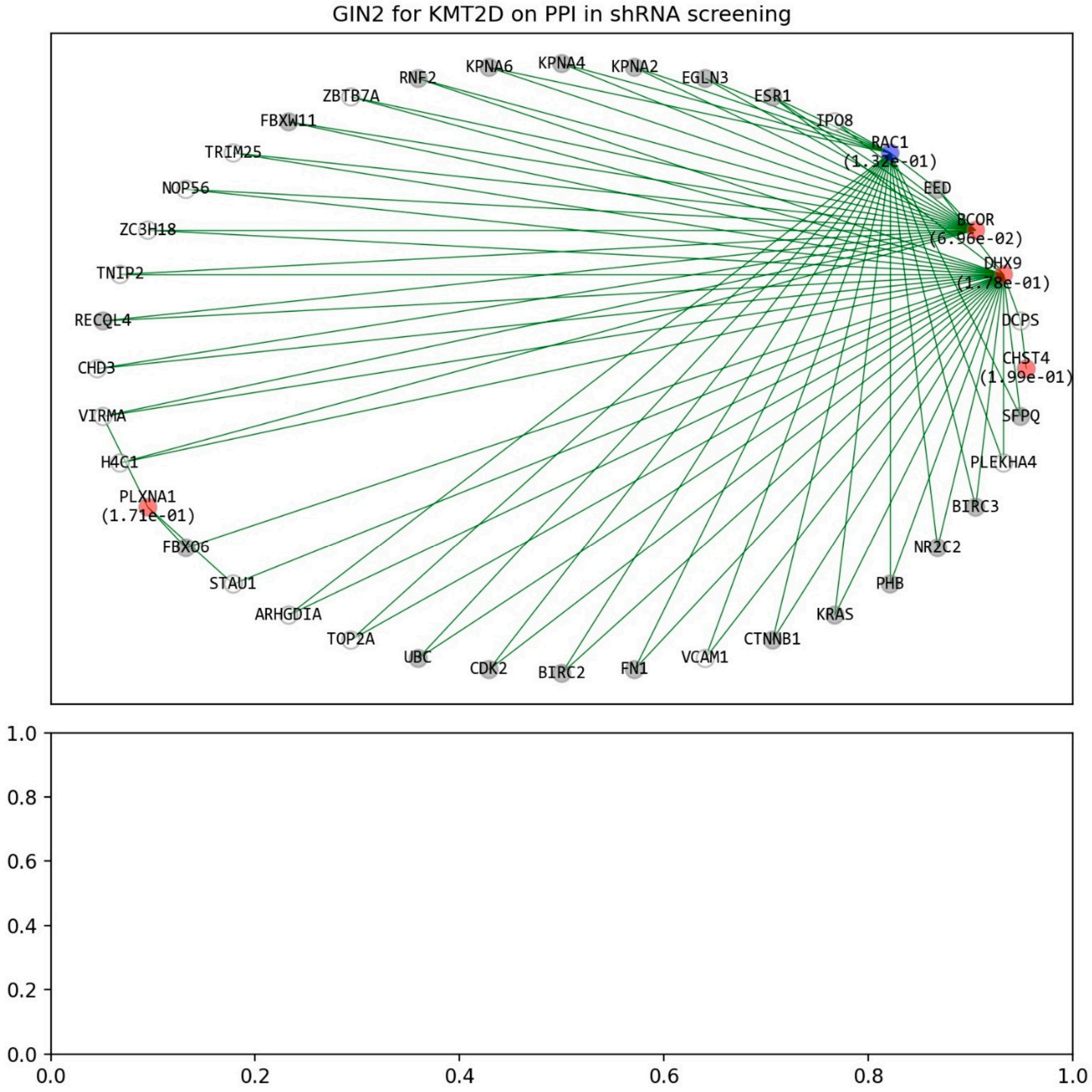

GIN2 for KMT2B on PPI in shRNA screening

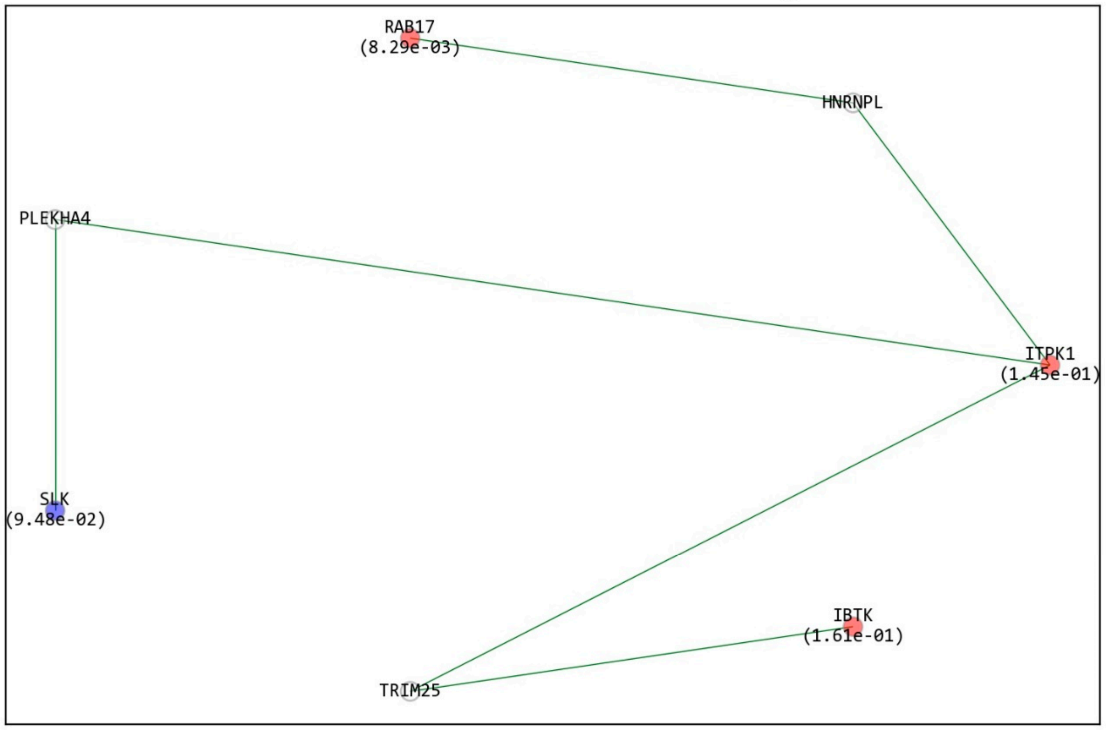

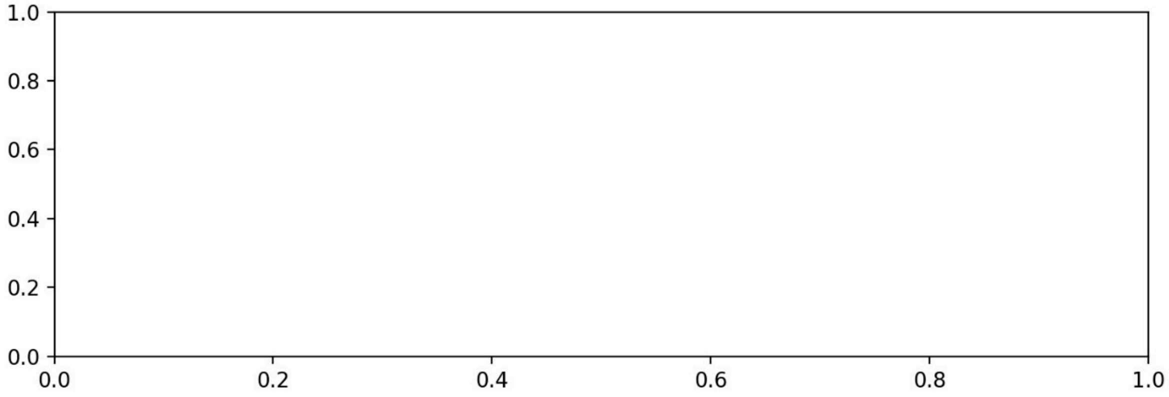

GIN2 for MYH9 on PPI in shRNA screening

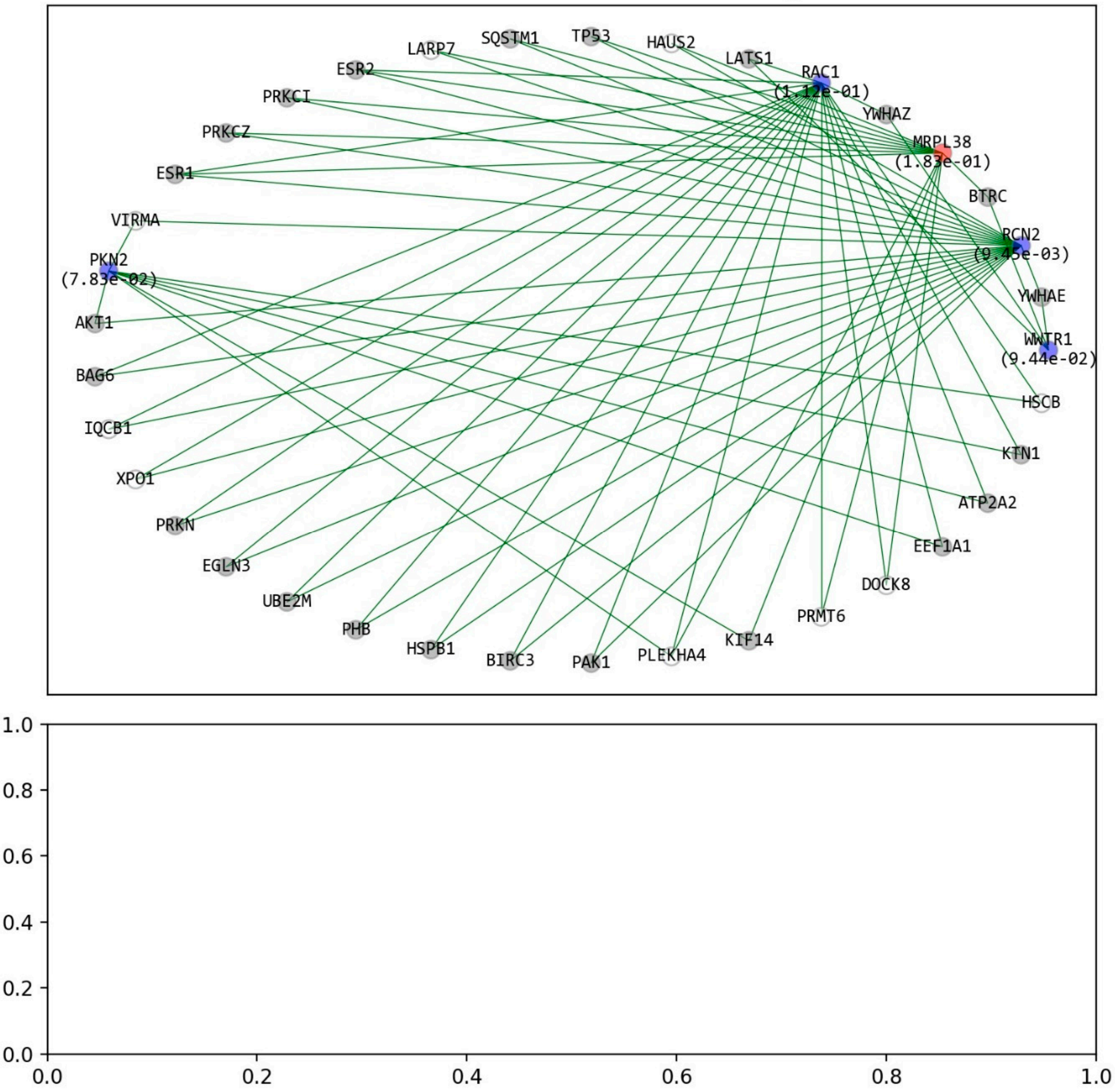

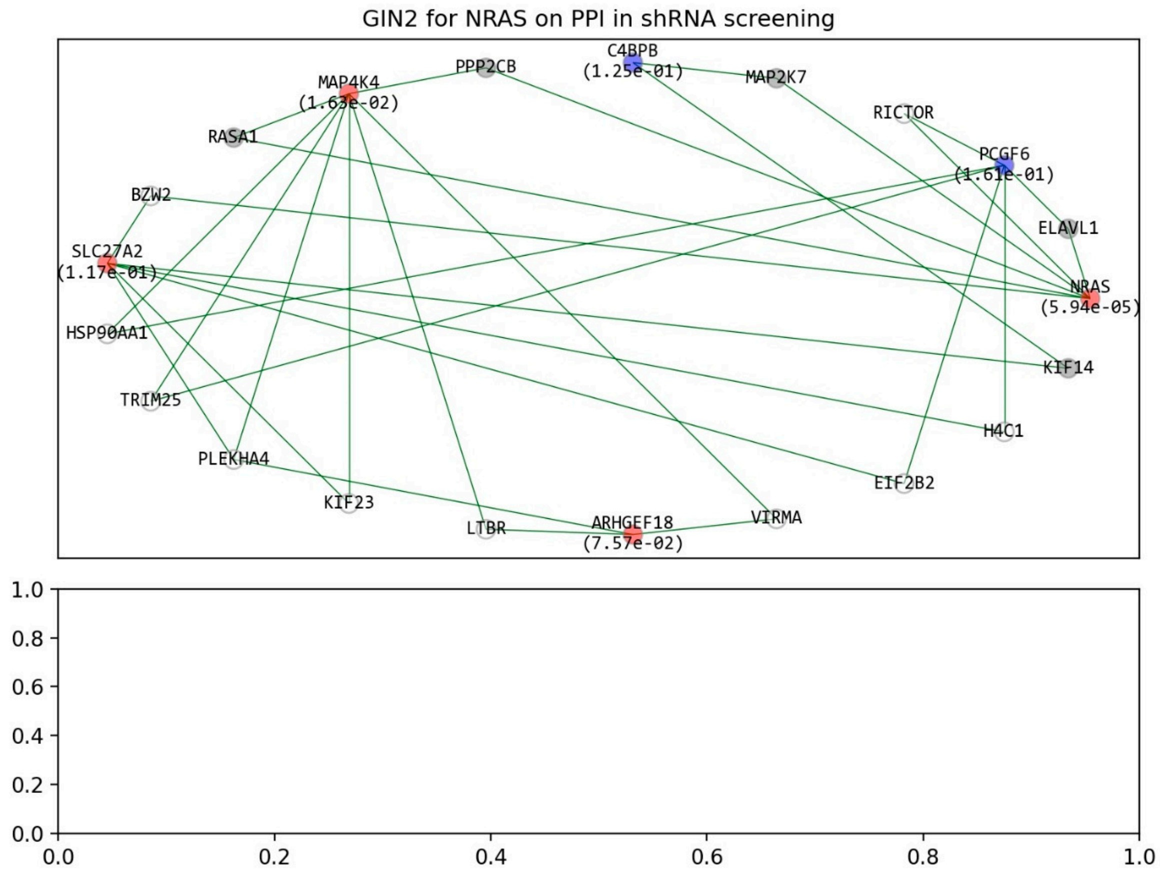
GIN2 for NRAS on PPI in shRNA screening
PPP2CB
C4BPB
(1.25e-01)
MAP2K7
MAP4K4
(1.63e-02)
RICTOR
RASA1
PCGF6
(1.61e-01)
BZW2
ELAVL1
SLC27A2
(1.17e-01)
NRAS
(5.94e-05)
HSP90AA1
KIF14
TRIM25
H4C1
PLEKHA4
EIF2B2
KIF23
VIRMA
ARHGEF18
(7.57e-02)
LTBR
1.0
0.8
0.6
0.4
0.2
0.0
0.0
0.2
0.4
0.6
0.8
1.0

GIN2 for PIK3CA on PPI in shRNA screening

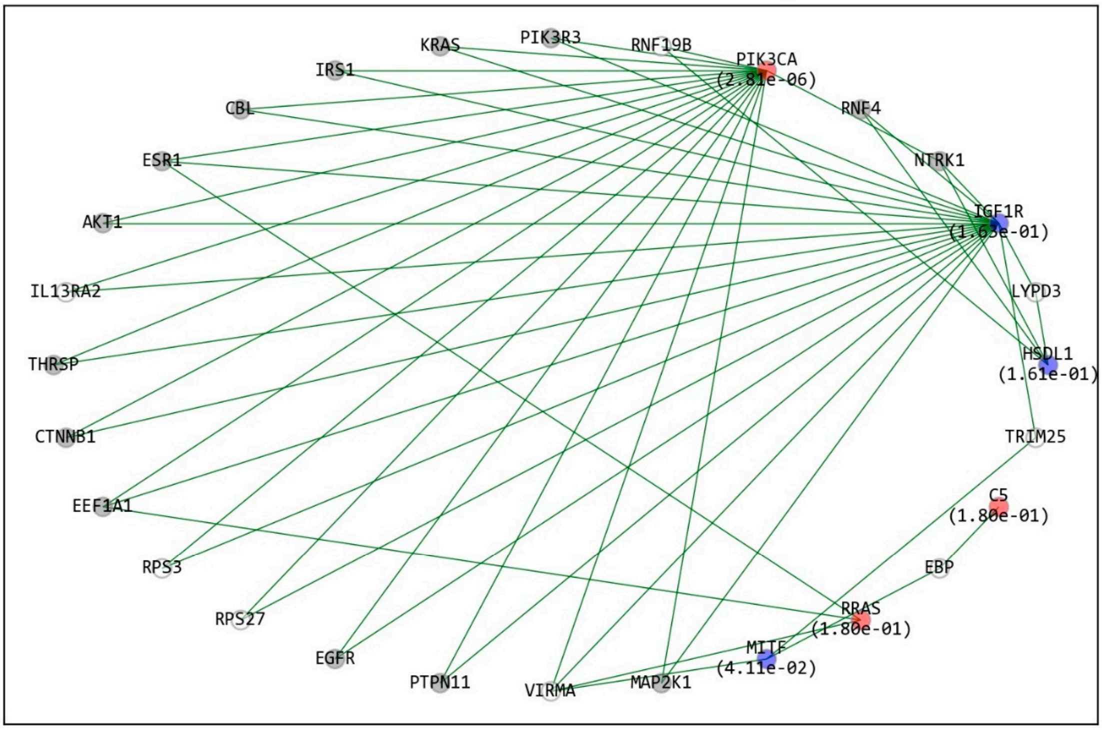

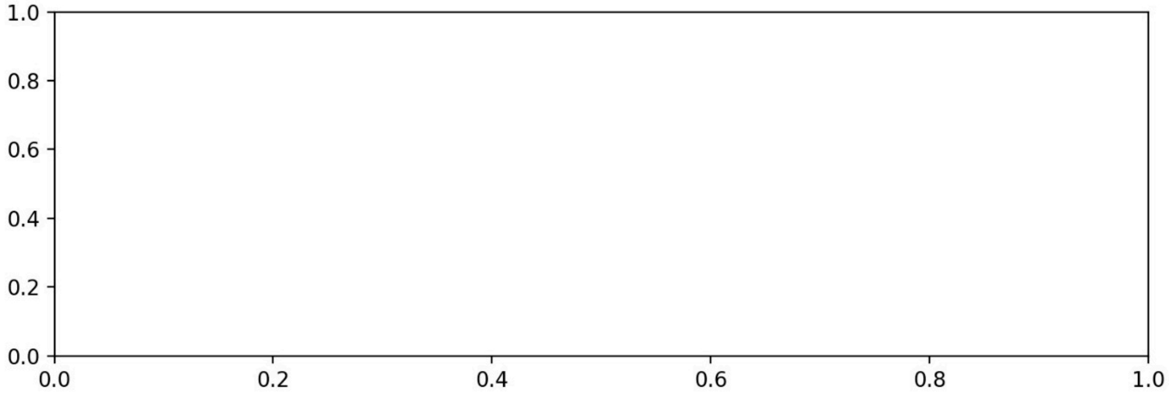

GIN1 for RB1 on PPI in shRNA screening

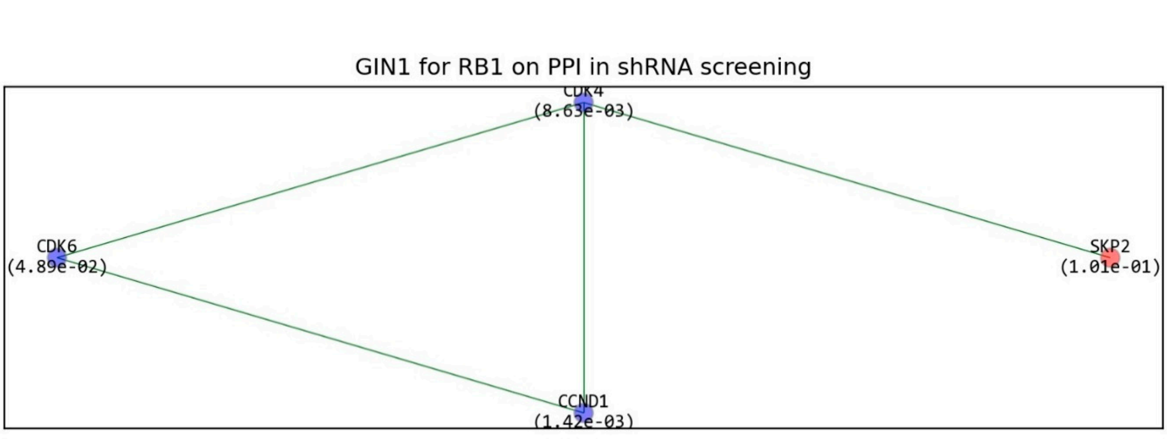

GIN1 for VHL on PPI in shRNA screening

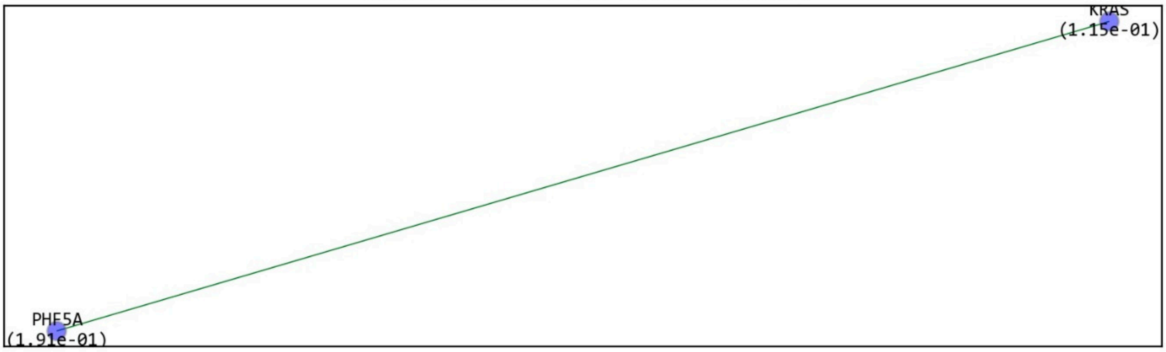

GIN1 for KRAS on PPI in shRNA screening

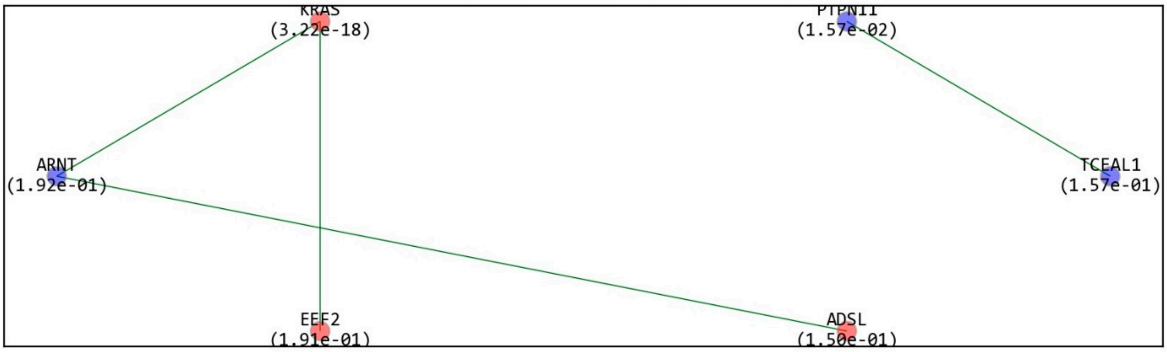

GIN1 for BRAF on PPI in shRNA screening

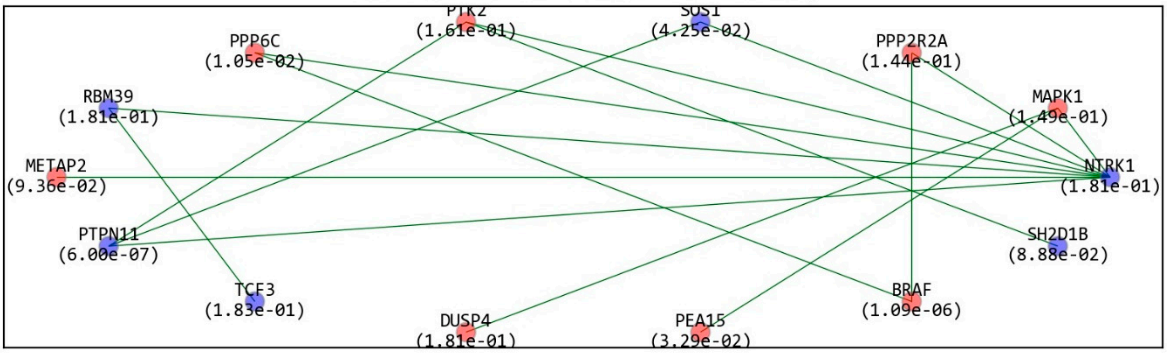

Figure S7. The sensitive GIs among the original GIs with exclusion procedure (SGWE) and without the exclusion procedure (SGOE) were compared to synthetic lethal interactions in MISL and synlethDB. The recall and precisio n measures are used.

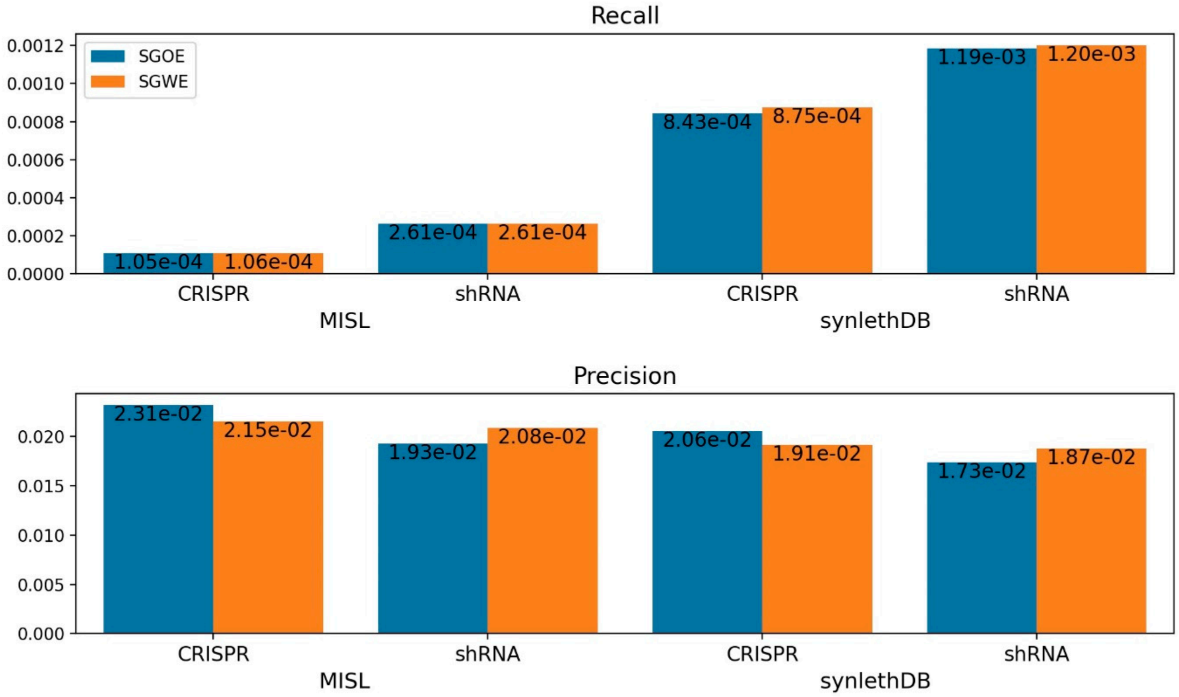

Figure S8. The recall of the sensitive GIs with no RP, RP2, and RP1. The sensitive GIs refined based on KEGG/PPI networks were evaluated with the SLIs in synlethDB and MISL. INIT: the initial sensitive GIs after mapping on the molecular networks (no RP was applied). RP2: the sensitive GIs after applying RP2 to the initial GIs. RP1: the sensitive GIs after applying RP1 to the initial GIs. RP: refining process. GI: genetic interaction.

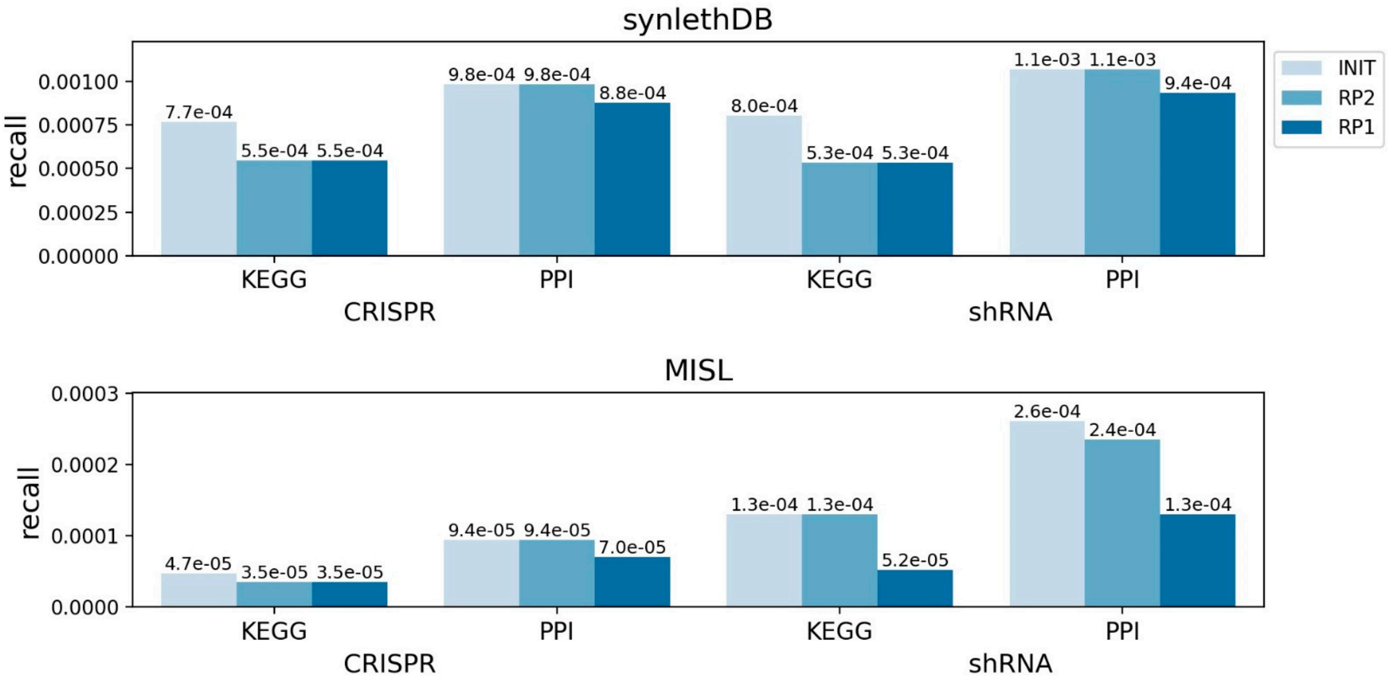

Figure S9. (a) the SPN1 on PPI for the mutated NRAS from CRISPR screening (b) the SPN1 on KEGG for the mutated NRAS from CRISPR screening.

(a)

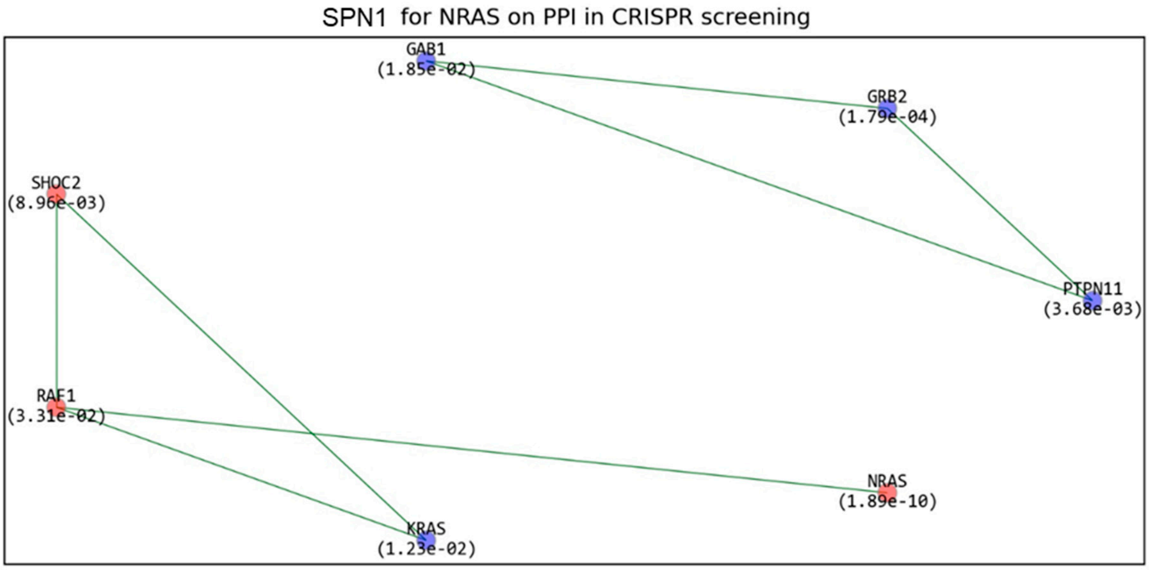

(b)

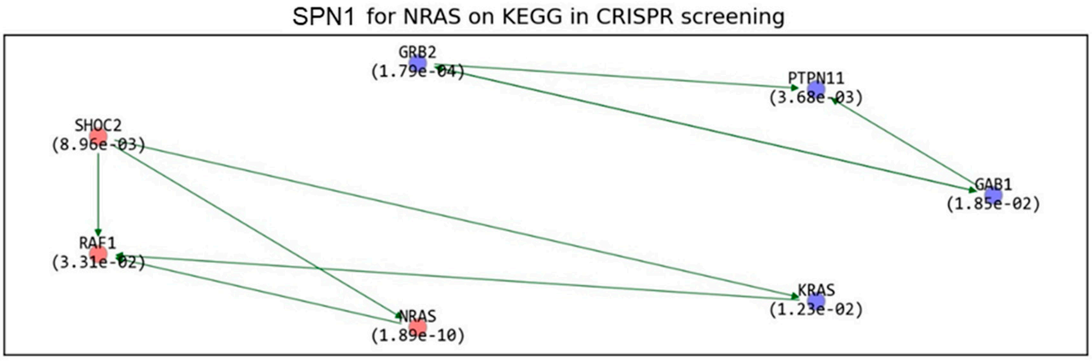

Figure S10. The number of cell lines for each cancer type in CRISPR and shRNA screening. The first bar represents the number of cell lines of the all considered cancer.

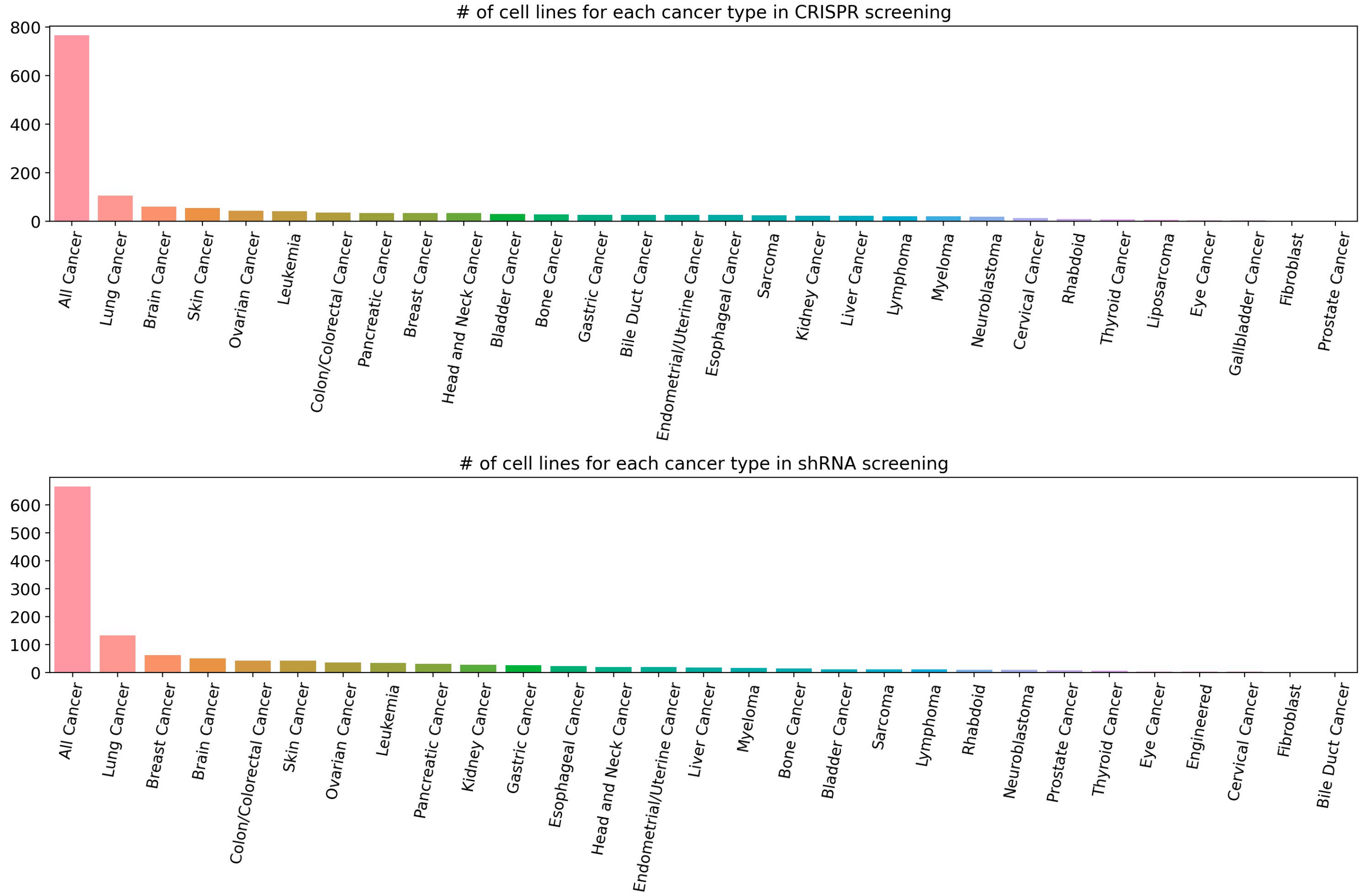

Supplement: Supplementary file 1 [file ijms-22-11114-s001.zip › Figure S1-S10.pdf]
